# Australia's Oldest Marsupial Fossils and their Biogeographical Implications

**DOI:** 10.1371/journal.pone.0001858

**Published:** 2008-03-26

**Authors:** Robin M. D. Beck, Henk Godthelp, Vera Weisbecker, Michael Archer, Suzanne J. Hand

**Affiliations:** School of Biological, Earth and Environmental Sciences, University of New South Wales, Sydney, New South Wales, Australia; University of Wisconsin, United States of America

## Abstract

**Background:**

We describe new cranial and post-cranial marsupial fossils from the early Eocene Tingamarra Local Fauna in Australia and refer them to *Djarthia murgonensis*, which was previously known only from fragmentary dental remains.

**Methodology/Principal Findings:**

The new material indicates that *Djarthia* is a member of Australidelphia, a pan-Gondwanan clade comprising all extant Australian marsupials together with the South American microbiotheres. *Djarthia* is therefore the oldest known crown-group marsupial anywhere in the world that is represented by dental, cranial and post-cranial remains, and the oldest known Australian marsupial by 30 million years. It is also the most plesiomorphic known australidelphian, and phylogenetic analyses place it outside all other Australian marsupials.

**Conclusions/Significance:**

As the most plesiomorphic and oldest unequivocal australidelphian, *Djarthia* may approximate the ancestral morphotype of the Australian marsupial radiation and suggests that the South American microbiotheres may be the result of back-dispersal from eastern Gondwana, which is the reverse of prevailing hypotheses.

## Introduction

Australia's marsupials are the most iconic members of the continent's fauna, but much remains unknown about their origins and early evolution. Current evidence suggests that the extant Australian marsupial orders evolved from an ancestor or ancestors that dispersed from South America, via Antarctica, sometime during the Late Cretaceous or early Palaeogene [1], [2], and that the orders diverged prior to the late Oligocene [2]. Recent phylogenetic analyses strongly support monophyly of Australidelphia [3]–[10], a pan-Gondwanan clade that includes all modern Australian marsupial orders as well as the South American microbiotheres (represented today by a single genus, *Dromiciops*). However, uncertain relationships within Australidelphia (notably, the position of the only extant South American australidelphian, *Dromiciops* [3]–[10], and doubts about the affinities of possible fossil australidelphians from South America [5], [11], mean that both the number and the direction of marsupial dispersals between South America and Australia are unclear.

The only pre-Oligocene Australian metatherians (marsupials and their stem-relatives) currently known are from a single site, the early Eocene Tingamarra fauna in southeastern Queensland [12]–[14]. The fossiliferous deposits at Tingamarra are green authigenic illite-smectite clays that appear to have formed in a shallow, low-energy aquatic environment [12], [15]. K-Ar dating of the illite gives a minimal age of 54.6±0.05 MYA ( = earliest Eocene) for the site [12]. Geological evidence and biocorrelative data from madtsoiid snakes [16], ‘graculavid’ birds [17] and an ‘archaeonycteroid’ bat [18] support the radiometric date (Text S1). Two Tingamarran metatherians have been described based on isolated teeth and mandibular fragments: the bunodont *Thylacotinga bartholomaii* [14] and the dilambdodont *Djarthia murgonensis* [13]. Neither can be confidently referred to a specific metatherian clade based on their preserved dental characters alone, so their relationship to the modern Australasian marsupial radiation and to the marsupial crown-group as a whole is unclear. Here we describe isolated petrosal and tarsal bones from Tingamarra that we refer to *Djarthia* on the basis of relative size, comparative morphology and abundance. This new material clarifies the phylogenetic relationships of *Djarthia* and provides significant new evidence regarding key aspects of Gondwanan marsupial evolution and biogeography.

## Results and Discussion

### Tingamarran Metatherian Petrosals

**Figure 1:**
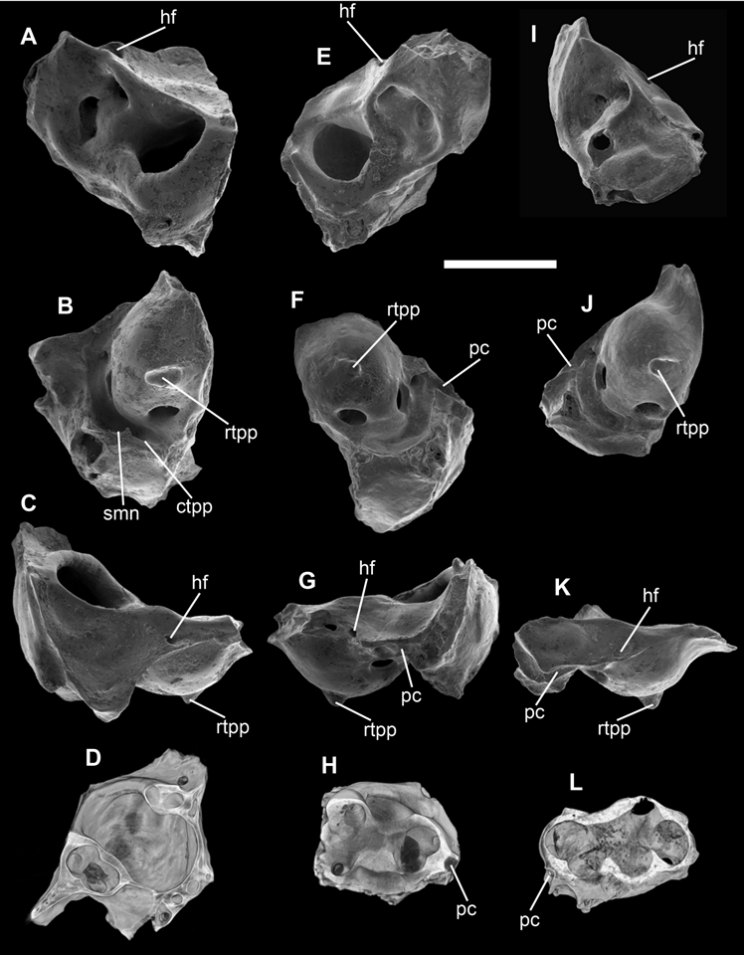
Isolated petrosals of *Djarthia murgonensis*. Specimens are illustrated by scanning electron micrographs of the cerebellar (A, E and I), tympanic (B, F and J), and squamosal (C, G and K) faces, and by coronal CT images (D, H and L). Scale bar, 2 mm. ctpp, caudal tympanic process of the petrosal; hf, hiatus fallopii; pc, prootic canal; rtpp, rostral tympanic process of the petrosal; smn, stylomastoid notch; Specimens illustrated (Queensland Museum palaeontology collection): A–D, QM F36393 (a right petrosal); E–H, QM F36397 (a left petrosal); I–L QM F32322 (a right petrosal).

Mammalian petrosals (which house the cochlea and semicircular canals) are highly complex bones that are commonly preserved in fossil deposits, and studies have identified numerous phylogenetically informative petrosal characters [6], [7], [19]–[21]. Seven isolated metatherian petrosals, representing a single morphotype, have been recovered from Tingamarra (Figure 1). They can be referred to Metatheria because they exhibit: cochlear coiling of >360 degrees (a therian synapomorphy [20]); presence in some specimens of a prootic canal (a mammalian plesiomorphy lost in all known eutherians except *Prokennalestes* [20] and ‘zhelestids’ [21]); absence of foramina or sulci for the internal carotid or stapedial arteries (loss of these being a synapomorphy of Metatheria [20], [22]). The hiatus fallopii (the exit for the greater petrosal nerve) opens dorsally, as in the Palaeogene North American metatherian *Herpetotherium* [8], Palaeocene South American metatherians [6], [7] and extant caenolestids [19] and marmosine didelphids [19], but unlike most australidelphians [23]; this feature may be plesiomorphic for crown-group Marsupialia. The rostral tympanic process of the petrosal is better developed than in deltatheroidans [22], most Late Cretaceous North American metatherians [19] and *Andinodelphys*, *Mayulestes* and *Pucadelphys* from the Middle Palaeocene of Bolivia [7], but resembles the condition in some Late Palaeocene metatherians from Brazil [6] and extant South American didelphids [19], [23] (with some exceptions, such as caluromyines [24]) and caenolestids [19], [23]; this feature may be a synapomorphy of crown-group Marsupialia. Within Australidelphia, *Dromiciops* (and the early Miocene microbiothere *Microbiotherium tehuelchum* [25]) dasyurids, diprotodontians and some peramelemorphians show considerable elaboration of either or both the rostral and caudal tympanic processes of the petrosal; the relatively simple structure of both of these processes in the Tingamarran petrosals is probably plesiomorphic within Australidelphia. A complete stylomastoid foramen within the caudal tympanic process for exit of the facial nerve (apomorphically present in dasyurids and macropodoids) is absent. A small, horizontal prootic canal, which transmits the lateral head vein [19], is present in three of the petrosals but absent in two others (Figure 1). The prootic canal is a mammalian plesiomorphy [20] retained by most stem-metatherians [19] but lost in some crown-group marsupials [23] including most australidelphians [23].

### Tingamarran Metatherian Tarsals

**Figure 2:**
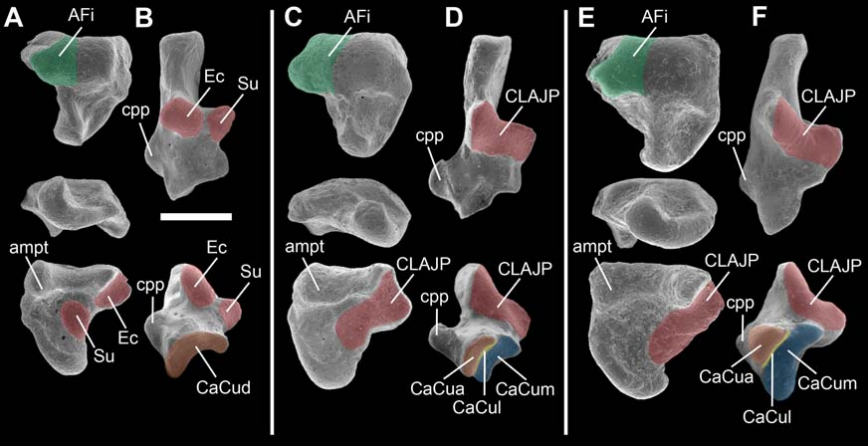
Comparison of isolated tarsals of *Djarthia murgonensis* with an extant australidelphian marsupial and an extant non-australidelphian (‘ameridelphian’) marsupial. Astragali (A, C, E) and calcanea (B, D, F) of the ‘ameridelphian’ didelphid *Thylamys elegans* (A–B), *Djarthia murgonensis* (C–D) and the australidelphian microbiotheriid *Dromiciops australis* (E–F). Astragali illustrated in dorsal (top), distal (middle) and ventral (bottom) views. Calcanea illustrated in dorsal (top) and distal (bottom) views. Scale bar, 1 mm. AFi, astragalofibular facet (green); ampt, astragalar medial plantar tuberosity; CaCua, auxiliary calcaneocuboid facet (orange); CaCud, distal calcaneocuboid facet (orange); CaCul, lateral calcaneocuboid facet (yellow); CaCum, medial calcaneocuboid facet (blue); CLAJP, continuous lower ankle joint pattern (red); cpp, peroneal process of the calcaneus; Ec, ectal facet (red); Su, sustentacular facet (red). Presence of the continuous lower ankle joint pattern and subdivision of the calcaneocuboid facet into three distinct facets are australidelphian synapomorphies [5]. Specimens illustrated (UNSW Palaeontology Laboratory collection (a–b, e–f) and Queensland Museum palaeontology collection): A–B, unregistered specimen; C, QM F52750; D, QM F52747; E–F, unregistered specimen.

Isolated tarsals are amongst the most commonly preserved mammalian post-cranial elements in fossil deposits, and the morphology of the tarsus-particularly the calcaneus and astragalus-has played a key role in our current understanding of metatherian phylogeny [5], [9]. Three isolated metatherian calcanea representing a single morphotype are known from Tingamarra (Figure 2C), as is a single metatherian astragalus that closely matches the calcanea in size and morphology of the conarticular joint surfaces (Figure 2D). Collectively, the Tingamarran specimens are clearly australidelphian because the ectal and sustentacular facets are fused on both the calcanea and astragalus, forming the diagnostic australidelphian ‘continuous lower ankle joint’ [5], and the calcaneocuboid facet of the calcanea is subdivided into three distinct facets (another synapomorphy of Australidelphia [5]; Figure 2C–F). These specimens are the oldest known that exhibit this distinctive morphology. Features that are probably plesiomorphic within Australidelphia include the gently rounded upper ankle joint surface of the astragalus (indicating that the upper ankle joint was extremely mobile, which suggests arboreality), a broad fibular facet of the astragalus (a possible apomorphy linking didelphids and australidelphians [5], [9]), a large astragalar medial plantar tuberosity that wraps under the sustentacular facet (absent in most australidelphians) and a large peroneal process of the calcaneus (greatly reduced in all other known australidelphians) [5], [9].

### Referral of Tingamarran Metatherian Petrosals and Tarsals to *Djarthia murgonensis*

We refer the petrosals and tarsals described here to *Djarthia murgonensis* because: 1) *Djarthia* is by far the most common dental taxon from Tingamarra, comprising ∼25% of all mammalian teeth from the site; 2) all metatherian petrosals and tarsals so far identified from Tingamarra each comprise a single morphotype; 3) regression analyses indicate that the sizes of these petrosals and tarsals correspond closely to those predicted for *Djarthia* based on dental measurements (Table S1, S2, Figure S1, S2).

### Phylogenetic Analysis and Molecular Divergence Dates

**Figure 3:**
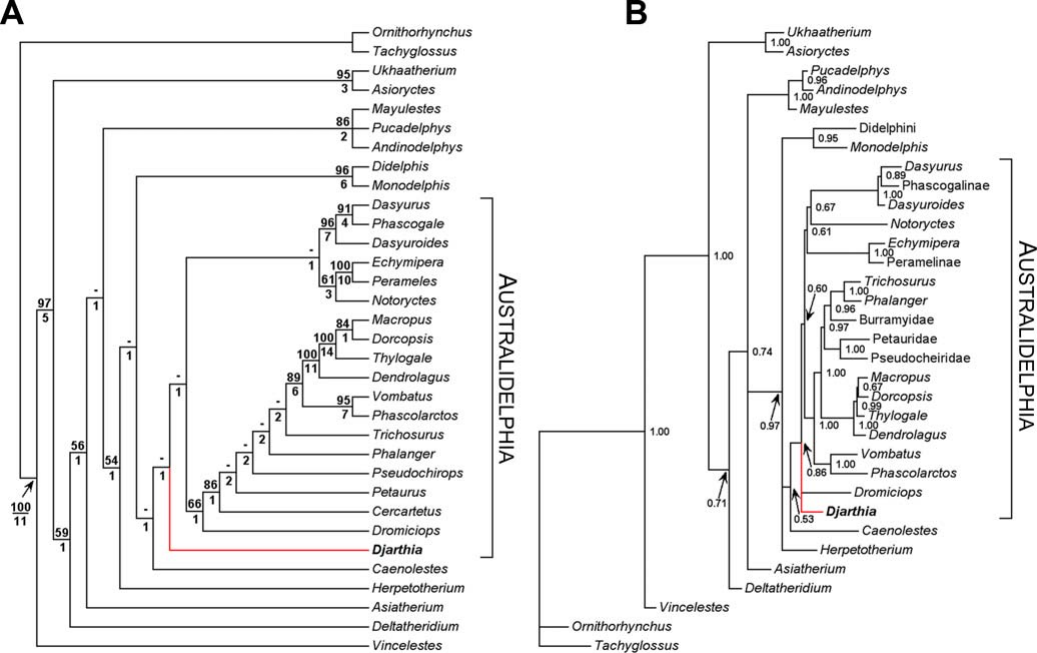
Phylogenetic relationships of *Djarthia murgonensis*. A, strict consensus of 2 most parsimonious trees (tree length = 886; consistency index (CI) excluding uninformative characters = 0.357; retention index (RI) = 0.646) from analysis of a 242 morphological character matrix [8]. Position of *Djarthia* highlighted in red. Australidelphia is indicated. Numbers above branches represent bootstrap values (2000 replicates); numbers below branches represent Bremer support values. B, Bayesian 50% majority rule consensus from analysis of the morphological matrix in combination with a 20.1 kb molecular data set [10]. Position of *Djarthia* highlighted in red. Australidelphia is indicated. Numbers at nodes represent Bayesian posterior probabilities.

Parsimony analysis of a 242 morphological character matrix [8] (Figure 3A, Text S2, S3) and partitioned Bayesian analysis of this matrix in combination with 20.1 kb of sequence data [10] (Figure 3B) confirm that *Djarthia* is a member of Australidelphia, but both analyses place *Djarthia* outside a clade comprising extant Australasian marsupials (Figure 3A–B). *Djarthia* is therefore the oldest known Australian crown-group marsupial by some 30 million years (over twice as old as the next oldest from Australasia [2]) and one of the oldest anywhere in the world. Divergence dates calculated using a Bayesian ‘relaxed molecular clock’ method [26] (Figure S3, Table S3) indicate that Australidelphia originated 65.0–75.1 MYA (95% CI = 59.2–84.3 MYA) and that the extant australidelphian orders diverged from each other 56.9–65.5 MYA (95% CI = 51.1–71.8 MYA). These dates are compatible with *Djarthia* as either a stem- or early crown-australidelphian. Because *Djarthia* appears to be more plesiomorphic than any other known australidelphian, it may approximate the ancestral morphotype of the Australasian marsupial radiation or of Australidelphia as a whole. The dentition of *Djarthia* indicates a generalised insectivorous diet [13], whilst the tarsal remains suggest scansorial or arboreal habits.

### Biogeographical Implications

*Khasia cordillerensis* [27] from early or middle Palaeocene (59.2–64.5 MYA) deposits at Tiupampa in Bolivia and *Mirandatherium alipoi* [28] from late Palaeocene (58.7–59.2 MYA) deposits at Itaborai in Brazil-both known only from dental specimens-have been referred to Microbiotheria, rendering them the oldest described australidelphians (although *Djarthia* is dentally more plesiomorphic than both of these taxa). However, these referrals have been questioned [5], [11] because they are based solely on characters of the dentition that are known to be highly homoplastic. Furthermore, no australidelphian-type tarsals have been found at either Tiupampa or Itaborai (even though tarsals of at least 13 metatherian taxa are known from the latter site [5]), and phylogenetic analyses of five different metatherian petrosal morphotypes from Itaborai indicate that none are referable to Australidelphia [6]. The oldest unequivocal South American australidelphian is *Microbiotherium tehuelchum*, which is from the early Miocene (16.2–16.6 MYA) Santa Cruz fauna of Argentina (roughly 40 million years younger than Tingamarra) and which exhibits distinctive microbiothere autapomorphies of the auditory region [25]. Possible non-australidelphian crown-group marsupials older than *Djarthia* include *Carolopaulocoutoia* (a possible paucituberculate [29]), isolated petrosals [6] and didelphid-like tarsals [5], all from Itaborai (which is approximately four million years older than Tingamarra). However, the affinities of these highly fragmentary taxa have yet to be investigated in the context of a broad-scale phylogenetic analysis that combines morphological and molecular sequence data. *Djarthia* is therefore the oldest crown-group marsupial known from dental, cranial and post-cranial remains, and the oldest with confidently resolved phylogenetic relationships; as such, it represents a robust calibration point for molecular dating analyses. The extremely plesiomorphic australidelphian morphology of *Djarthia* and the apparent absence of undoubted australidelphians from early Palaeogene deposits in South America raises the possibility that Australidelphia originated in Australia or elsewhere in eastern Gondwana, perhaps from a *Djarthia*-like ancestor. If so, Australidelphia did not originate in South America (as has usually been assumed [5], [9]) and the South American microbiotheres are the result of a later back-dispersal from eastern Gondwana. However, the early Palaeogene record of metatherians in South America is still relatively poorly known, particularly in the south of the continent; it is feasible that undoubted early Palaeogene South American australidelphians wait to be found. Possible microbiotheres have been described from the Middle Eocene La Meseta Formation of Seymour Island, but these taxa are known solely from isolated teeth and are approximately ten million years younger (and dentally more derived) than *Djarthia* [30].

## Materials and Methods

### Collection of fossils

All the fossil specimens described here were obtained by screen-washing of clay samples from the Tingamarra Local Fauna and subsequent microscope-assisted sorting of the concentrate.

### Justification for referral of the isolated Tingamarran metatherian petrosals and tarsals to *Djarthia murgonensis*

We base our referral of the isolated Tingamarran metatherian petrosals and tarsals to the Tingamarran metatherian *Djarthia murgonensis* (previously known only from dental specimens [13]) on the basis of: 1) relative abundance; 2) comparative morphology; 3) comparative size (using regression analyses).

*D. murgonensis* is by far the most common mammalian dental taxon at Tingamarra, comprising approximately 25% of all dental specimens. The second most common dental taxon is the bunodont metatherian *Thylacotinga bartholomaii* [14], which is considerably less common than *D. murgonensis* and is also approximately three times larger in linear dimensions (measurements taken from [14] and [13]) and is therefore far too large for the Tingamarran petrosals and tarsals described here (see Table S1, S2, Figure S1, S2). Other Tingamarran metatherians currently represented by dental specimens are far less common than *D. murgonensis* and *T. bartholomaii*. Thus, the Tingamarran petrosals and tarsals are likely to belong to *D. murgonensis* on the basis of relative abundance.

Collectively, the Tingamarran metatherian petrosals represent a single morphotype with minor variations in morphology, and are very similar in size (Figure 1). They can be referred to Metatheria based on cochlear coiling of >360° (a therian synapomorphy [20], [21]) and absence of sulci or foramina on the petrosal for the internal carotid or stapedial arteries (loss of these is a metatherian synapomorphy [22]). The petrosals most likely represent a plesiomorphic crown-group marsupial because of: 1) the absence of a groove on the anterior pole of the promontorium for the internal carotid artery (present in the South American stem-metatherians *Pucadelphys*, *Andinodelphys* and *Mayulestes* from the early or middle Palaeocene of Tiupampa in Bolivia and in some isolated petrosals from the late Palaeocene of Itaborai in Brazil [6], [7], [31]); 2) the presence of a well-developed rostral tympanic process of the petrosal (absent in *Pucadelphys*, *Andinodelphys* and *Mayulestes*) which is nevertheless not greatly enlarged as it is in some didelphids and many australidelphians; 3) no evidence of a complete stylomastoid foramen within the caudal tympanic process of the petrosal (this foramen is a derived feature of dasyurids and macropodoids); 4) loss in some specimens of the prootic canal (absence of this canal is common in crown-group marsupials, but apparently also occurred independently in borhyaenoids [23], [32]). Variation in the presence or absence of the prootic canal could potentially indicate that the petrosals described here represent more than one taxon. However, within marsupials polymorphism of this character has been reported at the family-level (caenolestids, didelphids, peramelids, peroryctids, dasyurids and phalangerids), genus-level (the dasyurid *Dasyurus*) and species-level (the didelphid *Philander opossum*) [23], [33]. The prootic canal has been described as present in *Dasyurus viverrinus* [33], but a *D. viverrinus* specimen from the University of New South Wales (AR6521) lacks an obvious prootic canal, indicating that this character is polymorphic within at least one australidelphian species. Although the prootic canal is apparently absent in adults of *Dromiciops gliroides*, it has been found in a late juvenile of this species, suggesting that this feature may be lost relatively late in ontogeny [23]. The polymorphism seen in the Tingamarran petrosals may reflect ontogenetic differences, or an intermediate stage in the loss or gain of the prootic canal. The Tingamarran petrosals cannot be unequivocally referred to Australidelphia because possible australidelphian synapomorphies of the petrosal [6], [31] show considerable polymorphism when a wider diversity of australidelphian taxa are considered [23].

Similarly to the petrosals, the Tingamarran metatherian tarsals represent a single morphotype with minor variations in morphology, and are also very similar in size (Table S2). They can be referred to Australidelphia because they exhibit fusion of the ectal and sustentacular facets, forming the australidelphian ‘continuous lower ankle joint pattern’, and subdivision of the calcaneocuboid facet into three distinct facets [5], [34]. The Tingamarran tarsals appear to be from a very plesiomorphic australidelphian because of the presence of a large peroneal process of the calcaneus (this process is reduced in all other known australidelphians [5]).

Based on its preserved dental features, *D. murgonensis* is probably a plesiomorphic member of the marsupial crown-group [13], although it could not be confidently assigned to either ‘Ameridelphia’ (a paraphyletic grade that includes the extant orders Didelphimorphia and Paucituberculata) or Australidelphia because of an apparent absence of unequivocal australidelphian dental synapomorphies [13]. Given that the Tingamarran metatherian petrosals and tarsals appear to represent a plesiomorphic crown-group marsupial and a plesiomorphic australidelphian respectively, referral of the petrosals and tarsals to *D. murgonensis* appears reasonable based on comparative morphology.

Following Szalay [5], Ekdale et al. [21] and Ladevèze [6], we have also used regression analyses to assess whether the Tingamarran metatherian petrosals and tarsals are of appropriate size for referral to *Djarthia murgonensis*. Ekdale et al. [21] and Ladevèze [6] calculated the area of the promontorium of the petrosal and molar area for a range of different eutherians and metatherians respectively, and used these in regression analyses. We have found that promontorium area is difficult to calculate unambiguously because the precise extent of the promontorium relative to adjacent regions of the petrosal is not always obvious, and it cannot be calculated in intact skulls of taxa in which the promontorium is not completely exposed in ventral view; instead, we have measured maximum petrosal length in ventral view for a range of different marsupial taxa (Table S1). We used mesiodistal length of the second upper molar (M2) as our dental measurement (Table S1), rather than molar area (as used by Ekdale et al. [21] and Ladevèze [6]), because the lingual portions of the M2 and M3 in the holotype of *D. murgonensis* are missing and so areas cannot be calculated for these teeth. Szalay [5] suggested that in metatherians the width of the lower ankle joint (i.e. combined width of the ectal and sustentacular facets of non-australidelphian taxa, or width of the continuous lower ankle joint of australidelphians) correlates with the mesiodistal lengths of the second upper and second lower molars, although he did not provide quantitative data to demonstrate this relationship. We therefore measured M2 mesiodistal length and lower ankle joint width (taken from the calcaneus) for a number of marsupials (Table S2) to investigate the allometric relationship between these measurements and to test the association of the Tingamarran metatherian tarsals with *D. murgonensis*. Measurements from specimens of a range of extant and fossil marsupials available at the University of New South Wales were taken using a Wild MMS235 measuring device. Graphs of 1) M2 mesiodistal length against petrosal maximum length (Figure S1), and 2) M2 mesiodistal length against lower ankle joint width were plotted (Figure S2), lines of best fit and their associated equations calculated and R2 values determined. Values for *D. murgonensis* were then plotted on each graph, assuming that the Tingamarran metatherian petrosals and tarsals are referrable to this taxon. Because all the Tingamarran metatherian petrosals referrable to *D. murgonensis* are incomplete, a composite measurement for maximum petrosal length was estimated from QM F36393, F36397 and F32322, which are three most complete specimens and are illustrated in Figure 1. Lower ankle joint width values for *D. murgonensis* were taken from QM F52747 (illustrated in Figure 2), F52748 and F 52749, which are all right calcanea. Of the dental specimens recovered from Tingamarra to date, those referable to *D. murgonensis* show the best fit in terms of size to the Tingamarran metatherian petrosals and tarsals, based on this regression analysis.

### Phylogenetic analyses

The morphological character matrix of Sánchez-Villagra et al. [8] is the most comprehensive currently available for marsupials, comprising 245 post-cranial, dental and cranial characters and including representatives of 14 extant families and the stem-metatherians *Deltatheridium*, *Asiatherium*, *Pucadelphys*, *Andinodelphys*, *Mayulestes* and *Herpetotherium*. *Djarthia* was added to this matrix, with scorings based on the petrosal and tarsal specimens described here and on dental specimens described by Godthelp et al. [13]. In the process of adding *Djarthia*, it became apparent that we have interpreted a number of morphological features differently to Sánchez-Villagra et al. [8], particularly those relating to the petrosal and related basicranial structures (characters 205–245). We have therefore scored a number of taxa differently for some characters, redefined other characters and excluded three characters that did not appear to us to comprise discrete states (characters 100, 212 and 237). These modifications are indicated in Text S2 and the modified matrix is given in Text S3. We have maintained the character numbers used by Sánchez-Villagra et al. [8] in their original matrix to faciliatate comparison with our revised matrix. Following Sánchez-Villagra et al. [8], the following morphological characters were ordered in all phylogenetic analyses: 4, 9, 10, 15, 16, 22, 30, 34–36, 39, 51, 53, 71, 74, 78, 79, 82, 83, 87, 93, 99, 101, 103–105, 109, 112, 118, 121, 123, 129, 134–138, 141, 146, 147, 149, 150, 152, 154, 168, 169, 174, 185, 188, 192, 202, 222 (see [8] and Text S2 for details). Following exclusion of characters 100, 212 and 237 (for the reasons given above and in Text S2), the resultant matrix comprised 242 characters scored for 33 taxa (Text S3).

The morphological character matrix was analysed using maximum parsimony as implemented in PAUP*4.0b10 [35]. The two-stage heuristic search used by Worthy et al. [36] was employed here. Support values were calculated using bootstrapping (2000 replicates using standard PAUP* settings) and Bremer support (using the two-stage heuristic search strategy of Worthy et al. [36]). The strict consensus of the most parsimonious trees, together with support values, is given in Figure 3a.

The morphological dataset was combined with the 20.1kb molecular supermatrix of Beck [10]-which comprises DNA sequence data from 7 nuclear genes (APOB, BRCA1, IRBP, PGK1, P1, RAG1, and VWF) and 15 mitochondrial loci (12S rRNA, 16S rRNA, tRNA valine, and 12 H-strand protein-coding genes)-and analysed using MrBayes 3.1.2 [37]. Further details regarding the supermatrix are given in [10]. Following Beck [10], the molecular supermatrix was partitioned by gene, codon position (for protein-coding genes) and stem and loop regions (for ribosomal genes), with each partition assigned the model selected for it by MrModelTest 2.2 [38] assuming the Akaike Information Criterion [39]. The morphological partition was assigned an Mk+G model [37], [40]. Using MrBayes 3.1.2, the combined analysis comprised four independent runs, each comprising 8 MCMC chains (7 ‘heated’ and 1 ‘cold’), with the temperature of the heated chains reduced from the default value of 0.2 to 0.15, to improve mixing. These analyses were run for 5 million generations, sampling trees every 100 generations. The first 4 million generations were discarded as burn-in, and a 50% majority rule consensus was constructed from the last one million generations (Fig. 3b).

### BEAST molecular dating analysis

Molecular divergence dates were calculated using the 20.1 kb molecular supermatrix of Beck [10] and the Bayesian relaxed molecular clock method implemented in BEAST 1.4 [26]. The partitioning scheme and models used in the MrBayes analyses (see above) were followed, and an uncorrelated lognormal relaxed clock [26] and a Yule tree prior (as recommended for species-level phylogenies [41]) were assumed. Prior estimates for the divergence dates for selected nodes were specified using transformed lognormal distributions [26], [41], [42]: these require specification of a ‘hard’ minimum bound (with a 0% probability of the divergence being younger than this date), a mean estimate and a ‘soft’ maximum bound (with a 5% probability of the divergence being older than this date). Here, the ‘hard’ minimum bounds were based on the minimum age of the oldest fossil that can be confidently assigned to a particular node. Given the incompleteness of the marsupial fossil record (particularly in Australasia), the mean estimates for divergence dates used here were taken from recent molecular studies. However, some current molecular dating methods may not be able to account for abrupt changes in the rate of molecular evolution, leading to overestimated divergence dates [43]; indeed, recent point estimates for divergences within mammals based on molecular data often appear unrealistically old from a palaeontological perspective (e.g. Wible et al. [44]; although the lower end of confidence intervals for these molecular divergences usually agrees well with the fossil record). For this reason, we selected lower bounds of estimated age ranges (usually one standard deviation less than the point estimate for a particular node) from previous molecular studies as mean values. The ‘soft’ maximum bound represents the oldest age for a divergence that, in our opinion, appears feasible based on current molecular and palaeontological evidence. The calibrations used are given in Text S4.

Because third codon positions of mitochondrial protein coding genes have been shown to mislead some phylogenetic analyses of marsupials[4], [10], two BEAST analyses were carried out: one using the full molecular matrix ( = ‘full’), and one with the third codon positions of the mitochondrial protein coding genes were excluded ( = ‘no mt3’). Following a pre-burnin of 1 million generations, both BEAST analyses were run for 10 million generations, sampling trees every 1000 generations. The first 9 million generations were discarded as burnin, with a 50% majority rule consensus constructed from trees sampled from the last 1 million generations.

The BEAST analyses supported the phylogeny given in Figure S3 (analyses of the ‘full’ and ‘no mt3’ datasets recovered the exact same topology except within macropodines-this conflict is represented as an unresolved trichotomy). As seen in Figure S3 (nodes 1 and 3), both BEAST analyses recovered a sister-group relationship between monotremes and marsupials ( = Marsupionta), which is almost certainly anomalous given that monophyly of marsupials and placentals ( = Theria) is now strongly supported by both morphological and recent molecular data [45]–[47]. However, relationships within marsupials are congruent with other recent molecular phylogenies [3], [4], [10], [48]. The divergence dates and 95% confidence intervals calculated for each node, using both the ‘full’ and ‘no mt3’ datasets, are given in Table S3. Australidelphian synapomorphies must have evolved between node 6 (the split between Australidelphia and Paucituberculata) and node 7 (the first divergence within Australidelphia), giving a range of 65.04–75.09 MYA (95% confidence interval = 59.21–84.32 MYA). Nodes 7, 8, 18 and 19 represent the divergences between the extant australidelphian orders; based on the results of the BEAST analyses, these occurred over the period 56.85–65.5 MYA (95% confidence interval = 51.09–71.77 MYA).

## Supporting Information

Text S1 Justification for an early Eocene age of the Tingamarra Local Fauna
(0.03 MB PDF)

Text S2 List of morphological characters scored for *Djarthia murgonensis* and/or modified from Sánchez-Villagra et al. [9]. Numbering follows Sánchez-Villagra et al. [9].
(0.06 MB PDF)

Text S3 Morphological character matrix
(0.02 MB PDF)

Text S4 Fossil calibration points used in the BEAST molecular dating analysis Figure S3 50% majority rule consensus from partitioned Bayesian analysis using BEAST (10 million generations, 9 million generation burn-in) of the ‘full’ and ‘no mt3’ versions of the molecular matrix of Beck [6]. Numbers correspond to nodes given in Table S3.
(0.04 MB PDF)

Table S1 Measurements of maximum petrosal length and M2 mesiodistal length for a range of extant and fossil marsupials (fossil taxa are indicated by †). Measurements for *Djarthia murgonensis* assume that the Tingamarran metatherian petrosals QM F36397, F36393 and F32322 (illustrated in Figure 1) are referrable to that taxon. No petrosal measurement is available for *Thylacotinga bartholomaii* because the petrosal of this taxon is currently unknown.
(0.02 MB PDF)

Table S2 Measurements of lower ankle joint width (taken from the calcaneus) and M2 mesiodistal length for a range of extant marsupials, plus *Djarthia murgonensis* and *Thylacotinga bartholomaii*. Measurements for *D. murgonensis* assume that the Tingamarran metatherian calcanea QM F52747 (illustrated in Figure 2), F52748 and F 52749 are referable to that taxon. No lower ankle joint width measurement is available for *T. bartholomaii*, as the calcaneus of this taxon is currently unknown.
(0.01 MB PDF)

Table S3 Molecular divergence dates within marsupials as calculated by BEAST assuming an uncorrelated lognormal relaxed clock. Dates were calculated using the supermatrix of Beck [6], including (‘full’) and excluding (‘no mt3’) the third codon positions of mitochondrial protein-coding genes. Node numbers correspond to the phylogeny in Figure S3. Point estimates and 95% confidence intervals are given for each node.
(0.01 MB PDF)

Figure S1 Plot of M2 mesiodistal length against maximum petrosal length for the specimens listed in Table S1. Specimens of *Djarthia murgonensis* and *Thylacotinga bartholomaii* are identified by squares and circles respectively. The predicted maximum petrosal length for *T. bartholomaii* was calculated according to the equation for the line of best fit.
(0.04 MB PDF)

Figure S2 Plot of M2 mesiodistal length against lower ankle joint width for the specimens listed in Table S2. Specimens of *Djarthia murgonensis* and *Thylacotinga bartholomaii* are identified by squares and circles respectively. Predicted lower ankle joint width for *T. bartholomaii* was calculated according to the equation for the line of best fit.
(0.04 MB PDF)

## References

[1] Woodburne MO, Case JA (1996). Dispersal, vicariance, and the late Cretaceous to early Tertiary land mammal biogeography from South America to Australia.. Journal of Mammalian Evolution.

[2] Archer M, Arena R, Bassarova M, Black K, Brammall J (1999). The evolutionary history and diversity of Australian mammals.. Australian Mammalogy.

[3] Amrine-Madsen H, Scally M, Westerman M, Stanhope MJ, Krajewski CW (2003). Nuclear gene sequences provide evidence for the monophyly of australidelphian marsupials.. Molecular Phylogenetics and Evolution.

[4] Phillips MJ, McLenachan PA, Down C, Gibb GC, Penny D (2006). Combined mitochondrial and nuclear DNA sequences resolve the interrelations of the major Australasian marsupial radiations.. Systematic Biology.

[5] Szalay FS (1994). Evolutionary history of the marsupials and an analysis of osteological characters..

[6] Ladevèze S (2007). Petrosal bones of metatherian mammals from the Late Palaeocene of Itaboraí (Brazil), and a cladistic analysis of petrosal features in metatherians.. Zoological Journal of the Linnean Society.

[7] Ladevèze S, Muizon C (2007). The auditory region of early Paleocene Pucadelphydae (Mammalia, Metatheria) from Tiupampa, Bolivia, with phylogenetic implications.. Palaeontology.

[8] Sánchez-Villagra MR, Ladevèze S, Horovitz I, Argot C, Hooker JJ (2007). Exceptionally preserved North American Paleogene metatherians: adaptations and discovery of a major gap in the opossum fossil record.. Biology Letters.

[9] Szalay FS, Sargis EJ (2001). Model-based analysis of postcranial osteology of marsupials from the Palaeocene of Itaboraí (Brazil) and the phylogenetics and biogeography of Metatheria.. Geodiversitas.

[10] Beck RMD (2008). A dated phylogeny of marsupials using a molecular supermatrix and multiple fossil constraints.. Journal of Mammalogy.

[11] Wroe S, Ebach M, Ahyong S, Muizon C, Muirhead J (2000). Cladistic analysis of dasyuromorphian (Marsupialia) phylogeny using cranial and dental characters.. Journal of Mammalogy.

[12] Godthelp H, Archer M, Cifelli RL, Hand SJ, Gilkeson CF (1992). Earliest known Australian Tertiary mammal fauna.. Nature.

[13] Godthelp H, Wroe S, Archer M (1999). A new marsupial from the Early Eocene Tingamarra Local Fauna of Murgon, southeastern Queensland: a prototypical Australian marsupial?. Journal of Mammalian Evolution.

[14] Archer M, Godthelp H, Hand SJ (1993). Early Eocene marsupial from Australia.. Kaupia.

[15] Salisbury SW, Willis PMA (1996). A new crocodylian from the early Eocene of southeastern Queensland and a preliminary investigation of the phylogenetic relationships of crocodyloids.. Alcheringa.

[16] Scanlon JD (2005). Australia's oldest known snakes: *Patagoniophis*, *Alamitophis*, and cf. *Madtsoia* (Squamata: Madtsoiidae) from the Eocene of Queensland.. Memoirs of the Queensland Museum.

[17] Boles WE (1999). Early Eocene shorebirds (Aves: Charadriiformes) from the Tingamarra Local Fauna, Murgon, Queensland, Australia.. Records of the Western Australian Museum Supplement.

[18] Hand S, Novacek M, Godthelp H, Archer M (1994). First Eocene bat from Australia.. Journal of Vertebrate Paleontology.

[19] Wible JR (1990). Late Cretaceous marsupial petrosal bones from North America and a cladistic analysis of the petrosal in therian mammals.. Journal of Vertebrate Paleontology.

[20] Wible JR, Rougier GW, Novacek MJ, McKenna MC (2001). Earliest eutherian ear region: a petrosal referred to *Prokennalestes* from the Early Cretaceous of Mongolia.. American Museum Novitates.

[21] Ekdale EG, Archibald JD, Averianov AO (2004). Petrosal bones of placental mammals from the Late Cretaceous of Uzbekistan.. Acta Palaeontologica Polonica.

[22] Rougier GW, Wible JR, Novacek MJ (1998). Implications of *Deltatheridium* specimens for early marsupial history.. Nature.

[23] Sánchez-Villagra MR, Wible JR (2002). Patterns of evolutionary transformation in the petrosal bone and some basicranial features in marsupial mammals, with special reference to didelphids.. Journal of Zoological Systematics and Evolutionary Research.

[24] Voss RS, Jansa SA (2003). Phylogenetic studies on didelphid marsupials II. Nonmolecular data and new IRBP sequences: separate and combined analyses of didelphine relationships with denser taxon sampling.. Bulletin of the American Museum of Natural History.

[25] Segall W (1969). The middle ear region of *Dromiciops*.. Acta Anatomica.

[26] Drummond AJ, Ho SYW, Phillips MJ, Rambaut A (2006). Relaxed phylogenetics and dating with confidence.. PLoS Biology.

[27] Marshall LG, Muizon C (1988). The dawn of the age of mammals in South America.. National Geographic Research.

[28] Paula Couto C (1952). Fossil mammals from the beginning of the Cenozoic in Brazil. Marsupialia: Didelphidae.. American Museum Novitates.

[29] McKenna MC, Bell SK (1997). Classification of mammals above the species level..

[30] Goin FJ, Case JA, Woodburne MO, Vizcaino SF, Reguero MA (1999). New discoveries of “opposum-like” marsupials from Antarctica (Seymour Island, Medial Eocene).. Journal of Mammalian Evolution.

[31] Ladevèze S (2004). Metatherian petrosals from the Late Paleocene of Itaboraí (Brazil), and their phylogenetic implications.. Journal of Vertebrate Paleontology.

[32] Muizon C de (1999). Marsupial skulls from the Deseadan (late Oligocene) of Bolivia and phylogenetic analysis of the Borhyaenoidea (Marsupialia, Mammalia).. Geobios.

[33] Sánchez-Villagra MR (1998). Patterns of morphological change in the ontogeny and phylogeny of the marsupial skull [Unpublished Ph. D. Dissertation]..

[34] Szalay FS, Archer M (1982). A new appraisal of marsupial phylogeny and classification.. Carnivorous marsupials.

[35] Swofford DL (2002). PAUP*: phylogenetic analysis using parsimony (*and other methods). 4.0b10 ed..

[36] Worthy TH, Tennyson AJD, Archer M, Musser AM, Hand SJ (2006). Miocene mammal reveals a Mesozoic ghost lineage on insular New Zealand, southwest Pacific.. Proceedings of the National Academy of Sciences of the United States of America.

[37] Ronquist F, Huelsenbeck JP (2003). MRBAYES 3: Bayesian phylogenetic inference under mixed models.. Bioinformatics.

[38] Nylander JAA (2004). MrModeltest v2. Evolutionary Biology Centre, Uppsala University: Program distributed by the author.

[39] Posada D, Buckley TR (2004). Model selection and model averaging in phylogenetics: advantages of the AIC and Bayesian approaches over likelihood ratio tests.. Systematic Biology.

[40] Lewis PO (2001). A likelihood approach to estimating phylogeny from discrete morphological character data.. Systematic Biology.

[41] Drummond AJ, Ho SYW, Rawlence N, Rambaut A (2007). A rough guide to BEAST 1.4.

[42] Ho SYW (2007). Calibrating molecular estimates of substitution rates and divergence times in birds.. Journal of Avian Biology.

[43] Kitazoe Y, Kishino H, Waddell PJ, Nakajima N, Okabayashi T (2007). Robust time estimation reconciles views of the antiquity of placental mammals.. PLoS ONE.

[44] Wible JR, Rougier GW, Novacek MJ, Asher RJ (2007). Cretaceous eutherians and Laurasian origin for placental mammals near the K/T boundary.. Nature.

[45] Kielan-Jaworowska Z, Cifelli RL, Luo Z-X (2004). Mammals from the age of dinosaurs: origins, evolution, and structure..

[46] van Rheede T, Bastiaans T, Boone DN, Hedges SB, de Jong WW (2006). The platypus is in its place: nuclear genes and indels confirm the sister group relation of monotremes and therians.. Molecular Biology and Evolution.

[47] Rougier GW, Martinelli AG, Forasiepi AM, Novacek MJ (2007). New Jurassic mammals from Patagonia, Argentina: a reappraisal of australosphenidan morphology and interrelationships.. American Museum Novitates.

[48] Phillips MJ, Pratt RC (2008). Family-level relationships among the Australasian marsupial “herbivores” (Diprotodontia: koala, wombats, kangaroos and possums).. Molecular Phylogenetics and Evolution.

