# Supplementary material for: Australia's Oldest Marsupial Fossils and their Biogeographical Implications
Source: PLoS One. 2008 Mar 26;3(3):e1858. doi: 10.1371/journal.pone.0001858 (PMC2267999; doi:10.1371/journal.pone.0001858)
Supplement: Text S1 — Justification for an early Eocene age of the Tingamarra Local Fauna (0.03 MB PDF) [file pone.0001858.s001.pdf]

TEXT S1: JUSTIFICATION FOR AN EARLY EOCENE AGE OF THE TINGAMARRA LOCAL FAUNA

Radiometric dating of illites from the mammal-bearing deposits at Tingamarra has given a minimum age of 54.6 +/- 0.05 million years, i.e. earliest Eocene [1]. However, it was subsequently suggested that the Tingamarra Local Fauna may in fact be late Oligocene or early Miocene, based on apparent mineralogical similarities to the late Oligocene-early Miocene Etadunna Formation [2]. This younger date has been discussed as a feasible alternative to an early Eocene age in a number of publications [2-5]. Although an Oligo-Miocene age for Tingamarra would not affect the hypothesis that *Djarthia murgonensis* is morphologically the most plesiomorphic known australidelphian, it would imply that *Djarthia* significantly post-dates the divergences of the Australasian marsupial orders and would greatly reduce the significance of the fauna as a whole: the Tingamarra Local Fauna would not represent the only known early Palaeogene Australasian mammal fauna; *Australonycteris clarkae* [6] would not represent one of the world's oldest bats; the Tingamarran passerines [7] would not be the oldest in the world by 25 million years; the Tingamarran metatherians would be similar in age to fossil marsupials from numerous Oligo-Miocene sites in Australia and considerably younger than the oldest known crown-group marsupials from South America.

We argue that an Oligo-Miocene date for the Tingamarra Local Fauna can be rejected, based on the following considerations. Firstly, radiometric dating of a superpositional basalt that forms the uppermost part of a >7m sequence of basalts interspersed with several paleosols (suggesting a considerable period of time between deposition of the Tingamarran fossils and formation of the dated basalt) gives an age of 29.0 +/- 0.02 million years, i.e. middle Oligocene [1], which is still considerably older than the estimated age of the Etadunna Formation (~25 Ma old). Secondly the sediments within the deposits that were originally described as "dolomitised" [1] have subsequently been shown by x-ray diffraction to be composed of calcium carbonate with no magnesium present, and hence are not dolomitic [8]; thus, Tingamarra is mineralogically unlike the late Oligocene-early Miocene central Australian deposits and there is no support for the hypothesis that they formed under similar palaeoclimatic conditions. Thirdly, several other early Palaeogene faunas and floras – e.g. the middle-late Eocene Rundle Formation [9], the early–middle Eocene Pomona Beds [10] and the Palaeocene-early Eocene Corinda Formation [11] - are

known from the same region that contains the Tingamarra Local Fauna. Finally, evidence from biocorrelation is congruent with an early Eocene age, rather than younger, because the Tingamarra Local Fauna includes: 1) the archaic bat *Australonycteris* [6], which, along with taxa such as *Icaronycteris*, *Archaeonycteris* and *Palaeochiropteryx*, represents a grade of primitive stem-bats that were globally extinct by the late Eocene [12,13]; 2) “graculavid” birds (a grade of plesiomorphic charadriiforms restricted to the Late Cretaceous-early Eocene, with a single possible exception from the early Oligocene [14]); 3) bunodont metatherians that may be confamilial with late Palaeocene-early Eocene South American taxa [15]; 4) madstoiid snakes that are congeneric with Late Cretaceous (~72 Ma old) South American taxa from the Los Alamitos Formation [16]. Furthermore, no diprotodontians (which should be easily identifiable based on the presence of an enlarged, procumbent lower incisor) are known from Tingamarra, whereas they are extremely diverse and well-represented at late Oligocene-early Miocene sites in Australia.

REFERENCES

1. Godthelp H, Archer M, Cifelli RL, Hand SJ, Gilkeson CF (1992) Earliest known Australian Tertiary mammal fauna. Nature 356: 514-516.
2. Woodburne MO, Case JA (1996) Dispersal, vicariance, and the late Cretaceous to early Tertiary land mammal biogeography from South America to Australia. Journal of Mammalian Evolution 3: 121-161.
3. Szalay FS (1994) Evolutionary history of the marsupials and an analysis of osteological characters. Cambridge: Cambridge University Press. 481 p.
4. Kemp TS (2005) The origin and evolution of mammals. Oxford: Oxford University Press. i-x, 1-331 p.
5. Rose KD (2006) The beginning of the age of mammals. Baltimore: Johns Hopkins University Press. i-xiv, 1-428 p.
6. Hand S, Novacek M, Godthelp H, Archer M (1994) First Eocene bat from Australia. Journal of Vertebrate Paleontology 14: 375-381.
7. Boles WE (1995) The world's oldest songbird. Nature 374: 21-22.

8. Salisbury SW, Willis PMA (1996) A new crocodylian from the early Eocene of southeastern Queensland and a preliminary investigation of the phylogenetic relationships of crocodyloids. Alcheringa 20: 179-226.

9. Buchanan L (2007) New Eocene crocodiles from Queensland: implications for mekosuchine evolution. In: Warren A, editor. Conference on Australasian Vertebrate Evolution, Palaeontology and Systematics 2007, Geological Society of Australia Abstracts 85. pp. 27-28.

10. Murphy PR et al. (1976) Geology of the Gympie 1: 250 000 Sheet Area. Geological Survey of Queensland, Report 96.

11. Cranfield LC et al. (1976) Geology of the Ipswich 1:250 000 Sheet Area. Geological Survey of Queensland, Report 95.

12. Gunnell GF, Simmons NB (2005) Fossil evidence and the origin of bats. Journal of Mammalian Evolution 12: 209-246.

13. Hand SJ, Beck RMD, Worthy TH, Archer M, Sigé B (2007) Australian and New Zealand bats: the origin, evolution and extinction of bat lineages in Australia. Journal of Vertebrate Paleontology 27: 86A.

14. Boles WE (1999) Early Eocene shorebirds (Aves: Charadriiformes) from the Tingamarra Local Fauna, Murgon, Queensland, Australia. Records of the Western Australian Museum Supplement 57: 229-238.

15. Sigé B, Sempere T, Butler RF, Marshall LG, Crochet J-Y (2004) Age and stratigraphic reassessment of the fossil-bearing Laguna Umayo red mudstone unit, SE Peru, from regional stratigraphy, fossil record, and paleomagnetism. Geobios 37: 771-794.

16. Scanlon JD (2005) Australia's oldest known snakes: *Patagoniophis*, *Alamitophis*, and cf. *Madtsoia* (Squamata: Madtsoiidae) from the Eocene of Queensland. Memoirs of the Queensland Museum 51: 215-235.
