# Supplementary material for: Australia's Oldest Marsupial Fossils and their Biogeographical Implications
Source: PLoS One. 2008 Mar 26;3(3):e1858. doi: 10.1371/journal.pone.0001858 (PMC2267999; doi:10.1371/journal.pone.0001858)
Supplement: Text S2 — List of morphological characters scored for Djarthia murgonensis and/or modified from Sánchez-Villagra et al. [9]. Numbering follows Sánchez-Villagra et al. [9]. (0.06 MB PDF) [file pone.0001858.s002.pdf]

82. Astragalus, angle between medial and lateral facets for tibia*: 0 = 90 degrees; 1 = intermediate; 2 = 180 degrees

***Djarthia*: 2**

83. Astragalonavicular facet extends on medial side of head: 0 = absent; 1 = medial.

***Djarthia*: 1**

84. Astragalonavicular facet extends on ventromedial area of head: 0 = absent; 1 = present.

***Djarthia*: 1**

85. Astragalus, dimensions of facet for navicular in distal view: 0 = transversely wider; 1 = dorsoventrally wider.

***Djarthia*: 0**

86. Astragalar medial plantar tuberosity (ampt) visibility in dorsal view: 0 = not visible; 1 = visible.

***Djarthia*: 1**

87. Astragalus, angle between facet for fibula and lateral facet for tibia*: 0 = 180 degrees; 1 = intermediate; 2 = 90 degrees.

***Djarthia*: 0**

88. Astragalar neck: 0 = absent; 1 = present.

***Djarthia*: 1**

89. Astragalus, relative width of head and neck: 0 = neck narrower or as wide as head; 1 = neck wider than head.

***Djarthia*: 1**

90. Astragalar sustentacular facet medial extent: 0 = does not reach medial edge of neck; 1 = reaches medial edge of neck.

***Djarthia*: 0**

91. Astragalonavicular facet position relative to facets for tibia: 0 = anterior to facets for tibia; 1 = medial relative to facets for tibia.

***Djarthia*: 0**

92. Astragalar canal: 0 = absent; 1 = present.

***Djarthia*: 0**

93. Posterior astragalocalcaneal facet major dimension orientation: 0 = straight; 1 = posteromedial to anterolateral.

***Djarthia*: 1**

94. Posterior astragalocalcaneal facet position in ventral view: 0 = extending up to posterior edge of astragalus; 1 = subterminal.

Djarthia: 0

95. Astragalus, ridge between medial and lateral astragalotibial facets: 0 = absent; 1 = present.

***Djarthia*: 0**

96. Astragalus, ridge between lateral astragalotibial and astragalofibular facets: 0 = absent; 1 = present.

***Djarthia*: 0**

97. Medial astragalotibial facet, posterior extent: 0 = does not reach posterior edge of lateral astragalotibial facet, 1= equal in anteroposterior length as lateral astragalotibial facet.

***Djarthia*: 0**

98. Astragalonavicular facet connection with sustentacular facet: 0 = absent; 1 = present.

***Djarthia*: 1**

99. Calcaneal sustentacular facet on sustentaculum*: 0 = no sustentaculum (facet is located in calcaneal body); 1 = on sustentaculum; 2 = facet is located above level of sustentaculum, which becomes the medial process.

***Djarthia*: 1**

100. Calcaneal sustentaculum position: 0 = subterminal; 1 = on anterior end.

Notes: it appears to us that the sustentaculum extends to the CaCu facet (i.e. to the anterior end of the calcaneus) even in taxa scored as '0' for this character by Sánchez-Villagra et al. [1], such as *Didelphis* and *Petaurus*. We therefore excluded this character from our analyses (it is scored as '-' for all taxa in the matrix below).

101. Ectal (or posterior calcaneoastragalar facet) longest dimension*: 0 = anteromedial to posterolateral; 1 = straight; 2 = posteromedial to anterolateral.

***Djarthia*: 0**

102. Calcaneal anterior peroneal tubercle: 0 = absent; 1 = present.

***Djarthia*: 1**

103. Calcaneal anterior peroneal tubercle shape: 0 = protuberance; 1 = laminar; 2 = process.

***Djarthia*: 1**

104. Calcaneal anterior peroneal tubercle position*: 0 = protruding anteriorly beyond calcaneocuboid facet; 1 = anterior, non-protruding; 2 = at a distance from anterior end of calcaneum.

***Djarthia*: 2**

105. Calcaneal sustentacular facet mesiolateral orientation: 0 = medial; 1 = dorsal. The sustentacular facet was scored as medial when at least the anterior area is directed medially, even if more posteriorly it curves to facing somewhat more dorsally.

***Djarthia*: 1**

106. Calcaneal sustentacular facet anteroposterior orientation: 0 = dorsal; 1 = 45 degrees dorsoanteriorly. This character was only coded for taxa having ch. 118 [1].

***Djarthia*: 1**

107. Calcaneal sustentacular facet anteroposterior convexity: 0 = concave or flat; 1 = convex.

***Djarthia*: 0**

108. Calcaneal plantar tubercle: 0 = absent; 1 = present.

***Djarthia*: 0 and 1**

Notes: The calcaneal plantar tubercle is very small but present in QM F52747 and F52748, but appears to be essentially absent in QM F52749.

109. Calcaneal sustentacular facet and posterior calcaneoastragalar facets merging*: 0 = separate; 1 = with narrow connection; 2 = merged.

***Djarthia*: 2**

110. Calcaneal sustentacular facet anterior edge: 0 = anterior to posterior facet; 1 = equal or posterior to posterior facet.

***Djarthia*: 0**

111. Calcaneal facet for fibula: 0 = absent; 1 = present.

***Djarthia*: 0**

112. Calcaneal facet for fibula orientation: 0 = lateral; 1 = dorsal.

***Djarthia*: -**

113. Calcaneal notch for cuboid pivot: 0 = absent; 1 = present.

***Djarthia*: 0**

114. Calcaneum sustentacular facet reaches anterior end: 0 = absent; 1 = present.

***Djarthia*: 0**

115. Calcaneum accessory facet anterior to sustentacular facet: 0 = absent 1 = present.

***Djarthia*: 0**

138. Number of upper molars*: 0 = four; 1 = three; 2 = none.

***Djarthia*: 0**

139. Upper molar M2 shape: 0 = triangular or semi-triangular; 1 = rectangular or semi-square.

***Djarthia*: 0**

140. Paracone and metacone placement in M2: 0 = medial or buccal; 1 = buccal margin.

***Djarthia*: 0**

141. Paracone versus metacone size in M2*: 0 = pa > me; 1 = pa equals to me; 2 = pa < me.

***Djarthia*: 2**

142. Centrocrista shape: 0 = linear; 1 = V-shaped; 2 = absent.

***Djarthia*: 1**

143. Metaconule: 0 = absent or not-well developed; 1 = well-developed or enlarged.

***Djarthia*: 1**

144. Trigonid versus talonid width: 0 = trigonid wider than talonid; 1 = trigonid subequal to talonid or trigonid narrower than talonid.

Comment: character refers to m2.

***Djarthia*: 1**

145. Paraconid on lower molars: 0 = absent; 1 = present.

***Djarthia*: 1**

146. Intersection of cristid obliqua with trigonid*: 0: absent; 1: lingual to protocristid notch; 2 below protocristid notch; 3: labial to protocristid notch.

***Djarthia*: 2**

Notes: Although three character states are listed by Sánchez-Villagra et al. [1], no taxon in their matrix is scored as '3'. Thus, although the cristid obliqua of *Djarthia* intersects the trigonid labial to the protocristid notch (i.e. state '3' of Sánchez-Villagra et al. [1]), we have scored it as state '2' to ensure consistency with the scorings of the other taxa.

153. Hypoconulid absent or present: 0 = absent; 1 = present.

***Djarthia*: 1**

155. Upper C: 0 = reduced or absent; 1 = caniniform or premolariform.

***Djarthia*: 1**

Notes: The character states given in the original list of Sánchez-Villagra et al. [1] have been swapped to match their codings in their matrix, and *Herpetotherium* is now coded as '1' as a large, caniniform upper canine is clearly present in this taxon [1].

156. Number of roots on upper canine: 0 = two; 1 = one.

***Djarthia*: 1**

158. Bunolophodonty or lophodonty developed: 0 = no; 1 = yes.

***Djarthia*: 0**

159. Selenodonty developed: 0 = no; 1 = yes.

***Djarthia*: 0**

205. Cavum supracochleare: is not roofed dorsally by the petrosal, and a depression in the anterior lamina for the geniculate ganglion is visible in dorsal view (0) or is entirely enclosed within the petrosal (1) or is not floored ventrally, such that there is no secondary facial foramen (2).

***Djarthia*: 1**

Notes: We have modified the original definition of this character given by Sánchez-Villagra et al. [1] – 'complete wall separating cavum supracochleare from cavum epiptericum: absent (0) or present (1)' - such that it is now scored on the basis of whether the cavum supracochleare (which contains the geniculate ganglion of the facial nerve) is entirely enclosed by the petrosal or not. In taxa scored as state '0', the petrosal does not roof the cavum supracochleare, and a large depression in the anterior lamina of the petrosal for the geniculate ganglion is visible in dorsal view [2]. In *Vombatus* [3] and *Deltatheridium* [4] the cavum supracochleare is not floored by the petrosal and hence no secondary facial foramen is formed – we have designated this distinctive morphology as an additional state. We are uncertain whether these character states form a clear morphocline, and therefore leave this character unordered.

206. Cavum epiptericum floored by: petrosal (0), petrosal and alisphenoid (1), primarily or exclusively by alisphenoid (2), primarily open as piriform fenestra (3).
***Djarthia*: 2 or 3**
Notes: Examination of sagitally-sectioned skulls indicates that *Dasyurus*, *Dendrolagus*, *Dorcopsulus*, *Vombatus* and *Phascolarctos* should be scored as state '2'. In *Pseudocheirus*, *Petaurus* and *Cercatetus* the petrosal makes a significant contribution to the floor of the cavum epiptericum, which we therefore score as state '1'.

207. Fossa subarcuata: deep (0) or extremely shallow (1)
***Djarthia*: 0**
Notes: This character was originally defined by Sánchez-Villagra et al. [1] as 'fossa subarcuata: smaller than its aperture (i.e., conical shape) (0) or larger than its aperture (i.e., spherical shape) (1)'. However, examination of the petrosals of a wide range of marsupial taxa indicates to us that the fossa subarcuata is always larger than its aperture, if only slightly. The size of the fossa relative to its aperture also appears to vary continuously. For these reasons, we have redefined this character so that it now reflects the depth of the fossa subarcuata, which is deep in all taxa considered here except *Vombatus* and *Tachyglossus*, in which it is only a very shallow depression.

208. Pars mastoidea: not extensively pneumatised (0) or extensively pneumatised and composed of cancellous bone (1)

***Djarthia*: 0**

Notes: This character was originally defined by Sánchez-Villagra et al. [1] as 'posterior exposure of the pars mastoidea: dorsoventrally elongated and approximately flat (0) or rounded and bulbous due to the excavation of the fossa subarcuata (1)'. However, we failed to see consistent differences in the shape of the mastoid exposure in taxa scored by Sánchez-Villagra et al. [1] as '0' (e.g. *Didelphis* and *Perameles*) with those scored as '1' (e.g. *Dasyurus*). Furthermore, the rounding of pars mastoidea in *Phalanger* and *Trichosurus* is not due to 'excavation of the fossa subarcuata', as the pars mastoidea forms a 'wedge' well separated from the pars canalicularis in phalangerids [5]. For these reasons, we have redefined this character so that it now distinguishes between the relatively unpneumatised pars mastoidea of most mammals (state '0') and the extreme pneumatisation of this region (which is largely composed of cancellous bone as a result) seen in *Trichosurus*, *Phalanger*, *Petaurus*, and *Pseudocheirus* (state '1').

209. Expansion of the crista petrosa into a salient crest that may cover the anterolateral part of the fossa subarcuata: absent (0) or present (1)

***Djarthia*: 0 and 1**

Notes: We have slightly altered the definition of this character – originally 'expansion of the crista petrosa that forms a thin lamina covering the anterolateral part of the fossa subarcuata: absent (0) or present (1)' - and rescored it to reflect the fact that the crista petrosa can be greatly hypertrophied (in e.g. *Trichosurus*) without necessarily covering part of the subarcuate fossa. In *Djarthia*, QMF36393 shows development of the crista petrosal so that it partially covers the subarcuate fossa, but QMF36397 does not, and it seems likely that, with increased taxon sampling, this character may prove to be polymorphic in other taxa.

210. Anterior lamina of petrosal exposure on the lateral wall of the braincase: present and large (0), or rudimentary (1), or absent (2)

***Djarthia*: 2**

211. Anterior lamina of petrosal: makes a major contribution to the medial wall of the middle cranial fossa (0) or makes a minor contribution (1)

***Djarthia*: 0**

Notes: Examination of a number sagittally sectioned-skulls shows that the anterior lamina of several marsupials forms a significant part of the middle cranial fossa, whereas in others the anterior lamina is makes a much less extensive contribution to the internal wall of the braincase. We have therefore modified Sánchez-Villagra et al.'s [1] original character – 'anterior lamina of petrosal: shows a large depression that may have received a part of temporal lobe of the brain and the posterior part of the trigeminal ganglion (0) or shows no depression (1)' - to reflect this. The relative contribution of the anterior lamina to the medial wall of the braincase can sometimes be determined from isolated petrosals as the degree of overlap by the alisphenoid is indicated by ridge on the anterior lamina (e.g. in *Dorcopsulus*). As there is no such ridge on the anterior lamina of *Djarthia*, we have scored it as '0'.

212. Internal acoustic meatus and fossa subarcuata: subequal and separated by a sharp wall (0), internal acoustic meatus narrower than the opening of the fossa subarcuata and separated from the latter by a thick shelf of bone (1)

Notes: examination of multiple taxa suggests to us that this character varies continuously, with no evidence of discrete character states. We have therefore excluded it from our analysis analyses (it is scored as '-' for all taxa in the matrix below).

213. Internal acoustic meatus: with prefacial commissure at least 50% the width of the internal acoustic meatus (0) with prefacial commissure less than 50% the width of the internal acoustic meatus (1)

***Djarthia*: 1**

Notes: We have modified the description of this character from that of Sánchez-Villagra et al. [1] – 'internal acoustic meatus: deep with thick prefacial commissure (0) or shallow with thin prefacial commissure (1)' - to clarify the definition of character states.

214. Deep groove for internal carotid artery excavated on anterior pole of promontorium: absent (0) or present (1)

***Djarthia*: 0**

Notes: although Sánchez-Villagra & Wible [3] report that a faint groove is present on the on the anterior pole of the promontorium in *Vombatus*, we have followed these authors and Sánchez-Villagra et al. [1] in scoring *Vombatus* as 0 for this character.

215. Deep and large fossa for the tensor tympani muscle excavated on the anterolateral aspect of promontorium, creating a battered ventral surface of the promontorium: absent (0) or present (1)

***Djarthia*: 0**

216. Epitympanic wing of petrosal: absent (0) or present (1)

***Djarthia*: 1**

217. Epitympanic wing of petrosal: flat (0), or confluent with bulla (1)

***Djarthia*: 0**

218. Lateral flange: large and lateral to promontorium (0) or greatly reduced or absent (1)

***Djarthia*: 1**

219. Broad shelf of bone surrounding fenestra cochleae and making a separation between it and aqueductus cochleae: absent (0) or present (1)

***Djarthia*: 0**

220. Rostral tympanic process of petrosal: absent (0) or present as a distinct crest or erected process (1)

***Djarthia*: 1**

Notes: We follow Sánchez-Villagra and Wible [3] and restrict our definition of a rostral tympanic process of the petrosal to a process that is anteromedial to the fenestra cochleae (not anterolateral to the fenestra vestibuli, as stated by Sánchez-Villagra et al. [1]), and have rescored this character for all taxa for which we had specimens available.

221. Rostral tympanic process of petrosal: forms an anterolaterally directed wing, sometimes contacting the ectotympanic, that does not extend on the whole length of the promontorium (0), or that does extend on the whole length of the promontorium (1)

***Djarthia*: 0**

Notes: We do not follow Crosby and Norris's [6] suggestion that the rostral tympanic process of the petrosal of phalangerids extends anteriorly beyond the cupula cochleae; instead, the rostral tympanic process of phalangerids appears to be a relatively discrete structure immediately anteromedial to the fenestra cochleae, and so we have scored both *Trichosurus* and *Phalanger* as '0'.

222. Tympanic aperture of hiatus Fallopii*: dorsal (0), intermediate (1), or ventral (2)

***Djarthia*: 0**

223. Stylomastoid foramen formed by the caudal tympanic process of the petrosal: absent (0) or present (1)

***Djarthia*: 0**

Notes: *contra* Sánchez-Villagra et al. [1], a stylomastoid foramen is present in all extant phalangeridan diprotodontians, posteroventral to the external acoustic meatus [7]. However, in 'possums' the walls of the foramen are formed by the pars mastoidea, which is invariably broken off in isolated petrosals of these taxa. To ensure that this character can be scored consistently for isolated petrosals as well as more complete skulls, we have specified that the stylomastoid foramen must be formed by the caudal tympanic process of the petrosal (or fusion of the rostral and caudal tympanic processes, as may be the case in dasyurids) – the character was originally simply 'stylomastoid foramen: absent (0) or present (1)'. Under our revised definition, only dasyurids and macropodoids of the taxa considered here can be scored as state '1'.

224.Inferior petrosal sinus: intrapetrosal (0) or between petrosal, basisphenoid and basioccipital (1) or endocranial (2)

***Djarthia*: 1**

225. Mastoid exposure: contacts the parietal (0), does not contact the parietal (1), or greatly reduced, forming a dorsoventrally narrow band on the ventrolateral corner of the posterior face of the cranium (2)

***Djarthia*: ?**

Notes: The states originally defined for this character by Sánchez-Villagra et al. [1] – 'mastoid exposure: large (0), narrow (1), or reduced pars mastoidea, internal to the braincase and wedged between the squamosal and exoccipital (2)' – do not appear to form discrete clusters. We have therefore redefined this character to aid unambiguous scoring. We follow Flannery et al. [8] in specifying a separate state ('2') for the phalangerids *Trichosurus* and *Phalanger*, *contra* Murray et al. [9], who illustrate a dorsally extensive condition in *Trichosurus*. The character states of the revised character do not appear to represent a clear morphocline, and hence this character was not ordered.

226. Mastoid tympanic process: large and vertical (0), small, slanted, and nodelike, on the posterolateral border of the stylomastoid notch and continuous with squamosal (1), or indistinct to absent (2)

***Djarthia*: 2**

Notes: although the mastoid tympanic process is damaged in all specimens of *Djarthia*, it appears to be almost complete in QMF36397 and is no larger than that of e.g. *Perameles*, *Dasyurus*, *Didelphis* or *Monodelphis*, all of which were scored by Sánchez-Villagra et al. [1] as state '2'. We differ from Sánchez-Villagra et al. [1] in scoring the presence of a large, vertical mastoid tympanic process (state '0') in *Macropus* (as well as in the other macropodids, *Phascolarctos* and *Vombatus*).

227. Caudal tympanic process of petrosal: absent (0) or present (1)

***Djarthia*: 1**

228. Caudal tympanic process of petrosal: forms a small crest that does not wholly floor the postpromontorial sinus (0) or forms an expanded lamina that floors the postpromontorial sinus (1)

***Djarthia*: 0**

Notes: although the cranium of *Notoryctes* shows extensive fusion such that sutures are largely unidentifiable, we have assumed that the enclosed postpromontorial sinus of this taxon is due to expansion of the caudal tympanic process, as in all other marsupials with similarly enclosed sinuses.

229. Petrosal plate: absent (0) or present (1)

***Djarthia*: 0**

230. Fossa incudis and epitympanic recess: continuous (0) or separated by a distinct ridge (1)

***Djarthia*: 0**

Notes: examination of isolated petrosals of dasyurids suggests to us that the fossa incudis and epitympanic recess are no more separated than they are in didelphids. By contrast, in phalangeridan diprotodontians (i.e. macropodids and 'possums'), these fossae are clearly separated by a strongly-developed ridge. There is no true fossa incudis in either *Vombatus* or *Phascolarctos*, as the crus brevis of the incus attaches to 'the wall of the interosseous cleft between the petrosal and squamosal bones' [7]; hence, these two taxa have been scored as '-' for this character.

231. Petrosal crest: absent (0) or present (1)

***Djarthia*: 1**

Notes: We consider that the petrosal crest separates the anteriormost part of the epitympanic recess from the posteriormost part of the hypotympanic sinus, which can be formed in the alisphenoid, squamosal and/or petrosal. In *Djarthia*, the petrosal crest appears as a lateral continuation of the crista parotica, forming a weakly developed anterior wall for the epitympanic recess. *Contra* Sánchez-Villagra et al. [1], we identify a petrosal crest in the dasyurids *Dasyurus*, *Dasyuroides* and *Phascogale*. The condition in diprotodontians is particularly noteworthy: a strongly developed lateral crest (the 'lateral malleolar ridge' *sensu* Aplin [7]) separates the epitympanic recess from the petrosal contribution to the hypotympanic sinus.

232. Lateral process of the petrosal: absent (0) or present (1)

***Djarthia*: 1**

Notes: We have modified this character from that originally defined by Sánchez-Villagra et al. [1] – ‘petrosal contribution to the lateral wall of the epitympanic recess: absent (0) or present (1)’ - because in some diprotodontians (notably macropodoids) the lateral wall of the epitympanic recess is formed entirely by the squamosal; however, in these taxa a large, ‘lateral process of the petrosal’ (*sensu* Aplin [7]) is present lateral to the petrosal part of the hypotympanic sinus. This lateral process appears to be homologous to the part of the petrosal forming the lateral wall of the epitympanic process in other marsupials. We therefore score this character and character 233 based on the condition of this ‘lateral process’.

233. Lateral process of the petrosal: massive, large shelf of bone, sometimes rounded (0), slender and triangular (1), or forming a thin lamina (2)

***Djarthia*: 1**

Notes: As for the previous character, we redefine the original character of Sánchez-Villagra et al. [1] – ‘petrosal contribution to the lateral wall of the epitympanic recess: massive, large shelf of bone, sometimes rounded (0), slender and triangular (1), or forming a thin lamina (2)’ – to refer to the lateral process of the petrosal *sensu* Aplin [7], which, as we discussed above, does not necessarily form the lateral wall of the epitympanic recess.

234. Prootic canal: present (0) or absent (1)

***Djarthia*: 0 and 1**

Notes: *Djarthia* appears to be polymorphic for this character. Aplin [7] noted a ‘small, intraosseous canal’ in adult *Phascolarctos* that resembles the prootic canal of other taxa, but argued that in *Phascolarctos* this canal most likely transmits a branch of the tympanic nerve rather than the lateral head vein, and so is not a true prootic canal. However, as this conclusion required embryological evidence not available in fossils, and as the osseous relationships of the canal in *Phascolarctos* closely resemble those of the true prootic canal in other taxa, we score *Phascolarctos* as state ‘0’.

235. Imprint of the transverse sinus bifurcation on the petrosal: absent (0) or present (1)

***Djarthia*: 0**

Notes: We score this character based on the presence or absence of a deep notch in the dorsal border of the petrosal where the transverse sinus meets the petrosal.

236. Foramina on the sigmoid sinus and/or prootic sinus, apparently connecting both vessels (i.e., sigmoid sinus vein): absent (0) or present (1)

***Djarthia*: 1**

Notes: we score this character based on the presence or absence of large foramina within the sigmoid sinus that enter into the substance of the petrosal.

237. Vascular groove medially adjacent to prootic sinus sulcus, on the pars mastoidea (i.e., prootic sinus vein or connection): absent (0) or present (1)

***Djarthia*: 1**

Notes: we have excluded this character as we have found it impossible to score consistently, and because it may be redundant with character 236 (it is scored as '-' for all taxa in the matrix below).

238. Posttemporal sulcus on the squamosal surface of the petrosal: present (0) or absent (1)

***Djarthia*: 0**

239. Posttemporal notch/foramen: present (0) or absent (1)

***Djarthia*: 0 and 1**

Notes: a small postemporal notch appears to be present in QMF36393 but not QMF36397

240. Transpromontorial sulcus: present (0) or absent (1)

***Djarthia*: 1**

241. Sulcus for stapedial artery: present (0) or absent (1)

***Djarthia* : 1**

242. Cochlear coiling: absent or less than 300° (0), or fully coiled (more than 360°) (1)

***Djarthia*: 1**

Notes: *Djarthia* was scored as '1' based on evidence from broken specimens and CT scans.

243. Tympanic sinus formed in the lateral trough (or anterolateral expansion of the pars canalicularis): absent (0) or present (1)
***Djarthia*: 0**

244. Large squamosal contribution to the external wall of the auditory bulla: absent (0) or present (1)
***Djarthia*: ?**
Notes: examination of broken specimens indicates that, *contra* Sánchez-Villagra et al. [1], the squamosal makes a considerable contribution to the hypotympanic sinus in many marsupials. However, to aid the scoring of unbroken specimens, we redefine this character from that used by Sánchez-Villagra et al. [1] – '(hypo)-tympanic sinus formed by petrosal and squamosal: absent (0) or present (1)'. Of the taxa under consideration, only *Vombatus* and *Trichosurus* show state '1'. *Pucadelphys*, *Mayulestes* and *Andinodelphys* have been scored as '0', as they lack a true auditory bulla.

245. Large alisphenoid contribution to the external wall of the auditory bulla: absent (0) or present (1)
***Djarthia*: ?**
Notes: As for character 244, we redefine this character from that originally specified by Sánchez-Villagra et al. [1] – '(hypo)-tympanic sinus formed by alisphenoid and petrosal, or by alisphenoid only: absent (0) or present (1)' – so that it can be scored unambiguously in specimens with intact auditory bullae.

REFERENCES

1. Sánchez-Villagra MR, Ladevèze S, Horovitz I, Argot C, Hooker JJ, et al. (2007) Exceptionally preserved North American Paleogene metatherians: adaptations and discovery of a major gap in the opossum fossil record. Biology Letters 3: 318-322.
2. Ladevèze S (2004) Metatherian petrosals from the Late Paleocene of Itaboraí (Brazil), and their phylogenetic implications. Journal of Vertebrate Paleontology 24: 202-213.

3. Sánchez-Villagra MR, Wible JR (2002) Patterns of evolutionary transformation in the petrosal bone and some basicranial features in marsupial mammals, with special reference to didelphids. Journal of Zoological Systematics and Evolutionary Research 40: 26-45.
4. Rougier GW, Wible JR, Novacek MJ (1998) Implications of *Deltatheridium* specimens for early marsupial history. Nature 396: 459-463.
5. Norris CA (1994) The periotic bones of possums and cuscuses: cuscus polyphyly and the division of the marsupial family Phalangeridae. Zoological Journal of the Linnean Society 111: 73-98.
6. Crosby K, Norris CA (2003) Periotic morphology in the trichosurin possums *Strigocuscus celebensis* and *Wyulda squamicaudata* (Diprotodontia, Phalangeridae) and a revised diagnosis of the tribe Trichosurini. American Museum Novitates 3414: 1-14.
7. Aplin KP (1990) Basicranial regions of diprotodontian marsupials: anatomy, ontogeny and phylogeny [Doctor of Philosophy]. Sydney: University of New South Wales. 389 p.
8. Flannery TF, Archer M, Maynes J (1987) The phylogenetic relationships of living phalangerids (Phalangeroidea: Marsupialia) with a suggested new taxonomy. In: Archer M, editor. Possums and opossums: studies in evolution. Sydney: Surrey Beatty & Sons and the Royal Zoological Society of New South Wales. pp. 477-506.
9. Murray P, Wells R, Plane M (1987) The cranium of the Miocene thylacoleonid, *Wakaleo vanderleuri*: click go the shears - a fresh bite at thylacoleonid systematics. In: Archer M, editor. Possums and opossums: studies in evolution. Sydney: Surrey Beatty & Sons and the Royal Zoological Society of New South Wales. pp. 433-466.
