# Supplementary material for: Australia's Oldest Marsupial Fossils and their Biogeographical Implications
Source: PLoS One. 2008 Mar 26;3(3):e1858. doi: 10.1371/journal.pone.0001858 (PMC2267999; doi:10.1371/journal.pone.0001858)
Supplement: Text S3 — Morphological character matrix (0.02 MB PDF) [file pone.0001858.s003.pdf]

Ornithorhynchus 0011110020 110112000? 0010210100 101--01101 010-101000 200??011?(01) 0101100101 0110011001 -0000000-0 0(01)??1?000- 11010-0000 1101000000 1021101010 00100542?? ?-?????000 ---00----0 ?0??110000 000?010000 ?0110?0000 0?10000010 3010000010 0-1000-000 -202?00-0- 00-010010- 00000

Tachyglossus 1010110020 110020000? 0010210100 101--00101 010-101001 000011010(01) 0101100101 011001(01)001 -2011100-0 000000010- 11220-0001 1001?01000 1011101010 00102542?? ?-?????000 ---00----0 ?0??110000 000?010200 ?0110?0000 0?10000010 4010101000 0-0000-100 -202?20-0- 00-010010- 10000

Vincelestes 10010?1120 11?1121010 ?2?101???1 0??1100111 001101111? ???01??1?0 1?11?001?? 0???011011 12000000-0 011000011- 01110-0000 1101?????? ?????????? ????13100 (12)?00100??? 011210000? ?0?00010?0 ?00???0000 1001001200 ???0?????? ????0000?0 0-0000-000 -200000-00 00-0000000 00000

Ukhaatherium 0?0?1????? ??????1?10 1?0?0????? ???020?1?? 001?0?1?10 ?????????0 1011?001?? ?10?0?1??? ?111001101 011011011- 11?2100000 ??0??????? ?????????? ?????00100 00??1????? 00?21??000 ?00??????1 ??11?????? ?000?????0 ?????????? ?????3???? ?-???????0 -?????10?? ?????????1 01??1

Asioryctes 000?001010 0-?1001011 ?????????? ?????????? ?????????? 210???11?? ?????????? 0???01?0?0 ?11???1101 0???11??1- ?1?010?0?? ??0??????? 1???????10 ?????00100 000011???? 0?12100000 00???00011 0011??3?00 0000000110 ?????????? ?????3?0?2 ?-10?101?0 ???1?2???0 ???1???111 01?00

Deltatheridium ?????????? ?????????? ?????????? ?????????? ?????????? ?????????? ?????????? ?????????? ?????????? ?????????1- 0???0-???? 11???????? ?????????? ?????11000 001011?122 0?12101001 ?1???01??1 ??10???0?? ??00011??0 ?????????? ????210002 0-00010100 --01?11000 00-0000001 11000

Mayulestes 0001001100 ???????01? ??01?????1 0000111111 0110011111 ?????????1 101??011?? 1???011010 1000?10110 011000?11- 1111000100 11010?000? ?????????? ?????00000 2010120122 0012111001 01?11010?1 0010??1000 0100011?20 ?????????? ?????100?2 ?-?1110100 -?01111000 1101?????1 11100

Pucadelphys 0001001100 1??1101011 1?0??10??2 0000211111 0010011111 ?????????1 1011000100 100?011010 1000110110 001000?11- 1111000100 110101000? ?????????? ?????00000 2111120122 0112111001 0100111001 0010??1000 0100001200 ???0?????? ????1{12}0001 0-11110110 -001111000 11000{01}11{01}1 11100

Andinodelphys 00010011?0 ???01?1?11 ??0??1???2 ?0002?1111 0011011111 2???0??101 1011?01100 100?011??? ??00110110 001000?11- 1111000100 11010?000? ???1001000

?011?00000 2110120122 000201100? 01001100?1 0010??101? 0100001200 ???0??????
????1{12}000? 0-11110110 -001?11000 1100001111 11100

Didelphis 1(01)(01)1010(01)10 1001221011 (01)200010012 0001101111 0(01)1(01)011110 2110111100 101110(01)101 1100011021 1211000110 0010110(01)1-0112110100 0-11110000 1011011011 1000000000 2101121122 0112111001 0100110101 001001(12)011 1000211(12)00 0?00100001 1410120002 1-00010101 0101121000 1110100001 11001

Monodelphis (01)101011010 1001101011 1200010012 00002101(12)1 0110001110 2110111100 101110(01)101 1102011021 1111000110 001010011- 0101110100 0-11100000 1011011011 1010000000 2101121122 0112111001 0101110101 001001(12)011 0000211200 0??1100001 0410120002 1-10010101 0101121000 1110100001 11001

Caenolestes 00000110?0 1101(01)(012)1110 ?2??010012 0000021101 0010011110 2110100100 1011101101 1101011020 0011000110 001010011- 0112110100 1001?00110 00110?1000 0011010011 1011122122 121211-001 0101110201 0010011010 ?010211100 0000000101 0210020002 0-10010101 0001?21100 0110010111 11001

Dasyuroides (01)(01)00011000 1011001(01)11 0?01010012 0000220111 0010001110 202010110? 1011?00101 1101001020 0211110110 001011001- 00--110120 0-00110010 0011001100 0012011000 2100120122 0012111001 0101010211 0010011011 0000(02)10100 0?0000???0 030?120012 ?-10010101 1011021110 1121???111 11001

Dasyurus 11(01)1011010 10?1(12)(01)1(01)11 (01)201010012 000??21011 1(01)1(01)001110 2020101101 1011100101 1102011021 1211110110 011011011- 0101110120 ??00100010 0011001100 0012011000 2100120122 0112111001 0101010211 0010011011 000021(01)200 010000(01)10? 0300120012 1-00010101 1111021110 1111111(01)(01)1 11001

Phascogale 11?0011010 1011(01)(012)1111 0201010012 0000121121 0010001110 2020101101 1011100101 1101011021 1211110110 011011001- 0101110120 ??00110010 0011001000 0012011000 2100120122 0212111001 0101010211 001001101? ?000?10?00 0??000???? ?30?120012 0-00010101 1111021110 1111111111 11001

Notoryctes 00(01)011?0(01)2 ?????01?10 0100210003 0000201010 1011001010 20???001?0 0011100111 1100011200 -011010110 001011001- 00--111121 1100?13010 01?111?110 0000221000 2-001?0?22 0102110001 0000010201 0010002011 ?0000?0?00 1??0001?00 2100???0?2 ?-1001?1?1 1????211?? ???1?????1 11001

Echymipera 0001011000 11112(012)1110 1201211013 1100111130 0110001110 2011000000 1011001010 1000001000 -201002110 000011101- 20--110111 1100001000 1121000110 0100211000 2111121122 1112111001 1101110211 0010012011 (01)010201100 ??000????? ?3??120002 0-10010101 0201021000 0121011111 11001

Perameles 000001?000 11012(012)1?10 1201211013 1100011120 0010001110 201110000? 1011001010 1000001000 -201102110 000111101- 20--110011 1100011000 112(01)000100 0100201010 2111121122 1112111001 1101110211 0010012011 (01)010201200 0?0000(01)000 2310120012 0-10010101 1201021100 012100?111 11001

Dromiciops (01)00001?000 10111(12)1?11 0100010011 0001200111 0011000110 2020100102 1011100101 110?011021 1211000110 001000001- 0102100120 0-01?10210 10110?1011 0000000000 1001120122 1112110001 1100010201 00101020{01}1 ?000201200 1211100100 0210120012 1-10011101 1011121110 1121?1?111 11001

Trichosurus 1(01)01011000 1011121111 0100(12)10011 00012(02)0111 0011001110 2000101100 1011101101 1101011021 1211010110 001001001- 0101110120 0-01?10210 1011001011 0100022011 0011021100 120111-101 01110(01)1321 1111102010 0000211200 12?(01)111100 1111120112 1-10010101 0101221101 112(01)101011 11011

Phalanger 10010(01)1010 10101(012)1111 0100010011 0001200111 0011001110 1000101100 1011100101 1101010021 1211000110 001001011- 0101111120 0-01?10210 1011001011 0100022011 0011021111 120111-101 01110(01)1321 11111021(12)0 0000211200 121111???0 ?21?120102 0-10010101 000122110? 1121000?11 11001

Petaurus 1101011010 0-11011111 0100210011 00012001(01)1 0011001110 1100001101 1011000101 1101011021 1211010110 001000001- 0101101120 0-00010210 0011001011 0100022010 1011022111 100111-001 11010(01)0321 1111101010 ?000001200 1211111200 1211110102 1-10010101 0101121101 1121000111 11001

Pseudochirops 1002011000 1010211110 0100010012 0001200111 0011000110 1100??1100 1011100101 1102010021 1211000110 001001011- 0101111120 0-00010210 1011000011 0100023010 0111011102 121011-011 0101011321 1111102010 ?000011200 12?11112?0 1211110102 1-10010101 0101121101 1121(01)01011 11001

Cercartetus 00000?1010 0-?1111111 0100110011 000021?111 00110?111? 1?00101100 1011?00101 1100011021 1211000110 001000001- 0112101120 1100?10210 0011001011 0100023010 0011021111 121111-001 0101?1?321 111110203? 0000211100 1???1?1??0 121?110012 0-100101?1 0001021101 112100??11 11001

Macropus 1002001000 1010221111 0101010??3 0002210111 1111000110 0000001112 1111111111 0100011220 1000111110 101111002- 1102110101 1200112001 1000-01(01)2- -103223011 1011021022 10000?-101 0111111321 0011102120 1000(02)11201 1211101200 1211120012 1-10010101 1211101101 1121(01)000(01)1 11001

Dendrolagus 100100110(01) 1010221111 0101210013 0002220111 0111001110 0100000111 1111101111 0100011020 1100001110 101011002- 0102110021 1200102000 1000-

0102- -103123011 1011021022 110001-101 0111111321 0011102120 0000(02)11201 ?????0???? ?2??120012 1-00010101 0211101101 112110??01 11001

Dorcopsis 1002011000 1010221110 0001210013 0002200111 1111000110 0000000112 1111111110 0100011220 1000111110 101111002- 1102110101 1200112001 1000-0112- -103223011 1011021022 1(01)0001-101 0111111321 0011102120 ?000211201 ?????????? ?2??120012 1-10010101 0211101101 1120101001 11001

Thylogale 1002011000 1010221111 0100210013 0002220111 1111000110 0?00001112 1111111111 0100011220 1000101110 101111002- 1102110101 1200112001 1000-0112- -103223011 1011021022 100001-101 0111111321 0011102120 000021(01)(12)01 ????101?0? 1211??0012 1-00?????1 021110110? ???????11? 11?01

Vombatus (01)001010010 0-11121111 0100100012 0001220111 1111001110 (01)000101102 1011101101 0101010021 1111001110 001011111- 00--100120 ??01?10010 1011001011 0100043011 101101?000 0-000?-011 0111101(01)21 0012103021 00001?1100 1100(01)0?210 2111221002 0-10010100 --0110100- 11210(01)?1?1 11010

Phascolarctos 1002010010 0-00221110 0100100011 0001200101 0011001110 0000001110 1011101101 1101000221 1011001110 001011111- 00--100120 ??01?10011 1011001?11 0100023010 1111022000 021001-011 0011111201 0011102111 ?000111200 1??000??10 2111120012 1-10010100 -20100110- 1110000111 11001

Herpetotherium ?????????? ??????1??? ??0??????? ?????????? 0???0????? ?????????1 1?1??00101 110?011020 1001000110 001010111- 21110-0100 1101?????? ?????????? ?????00000 2101122122 001211000? ?10011?2?1 ????012??? ??002?1??0 ?????????? ????020002 1-10010101 0001?11100 1111100111 11001

Asiatherium ?????????? ?????????? ????0????? 0?????01?1 ?0????1??1 ?00??????0 1??1?0?1?? ?10?0?1??? ?????????? ?????????- ?????????? ?????????? ?????????? ?????????0 0011?2???? ??1?1??00? ?????????? ???0??(12)??? ????0?1??? ???0?????? ???????0?? ?-???11??1 0?0??1100? ?????????1 ??0??

Djarthia ?????????? ?????????? ?????????? ?????????? ?????????? ?????????? ?????????? ?????????? ?211010110 001000011- 0112110(01)20 0-0?0????? ?????????? ???????000 211112???? ??1?11?00? ?????????? ?????????? ?????????? ?????????? ????1{23}00(01)2 0-10010101 0001?21000 112(01)0110?1 110??
