# Supplementary material for: Australia's Oldest Marsupial Fossils and their Biogeographical Implications
Source: PLoS One. 2008 Mar 26;3(3):e1858. doi: 10.1371/journal.pone.0001858 (PMC2267999; doi:10.1371/journal.pone.0001858)
Supplement: Text S4 — Fossil calibration points used in the BEAST molecular dating analysis Figure S3 50% majority rule consensus from partitioned Bayesian analysis using BEAST (10 million generations, 9 million generation burn-in) of the ‘full’ and ‘no mt3’ versions of the molecular matrix of Beck [6]. Numbers correspond to nodes given in Table S3. (0.04 MB PDF) [file pone.0001858.s004.pdf]

Divergence	Calibration
**Monotremata-Theria** (= 'root height')	Hard minimum = 162.5 MYA – age of oldest known theriimorph mammals (following Benton and Donoghue [1])<br><br>Mean = 185 MYA - lower end of the molecular estimates of van Rheede et al. [2]<br><br>Soft maximum = 220 MYA – the age of the oldest known mammals [3]
Ornithorhynchus-Tachyglossus	Hard minimum = 15.92 MYA – age (lower bound) of the oldest known tachyglossid, *Megalibgwilia* [4,5] (the apparent early Palaeocene ornithorhynchid *Monotrematum sudamericum* was not used to date this node, as it is possible that an ornithorhynchid-like morphology is plesiomorphic for crown-group monotremes as a whole)<br><br>Mean = 52.2 MYA – lower end of molecular date of Bininda-Emonds et al. [6]<br><br>Soft maximum = 125 MYA – age (lower bound) of oldest known monotreme, *Teinolophus trusleri* [7]
**Eutheria-Metatheria**	Hard minimum = 124.6 MYA – age (lower bound) of the oldest known eutherian, *Eomaia scansoria* [8], and the oldest known metatherian, *Sinodelphys szalayi* [9]<br><br>Mean = 165 MYA – lower end of the molecular estimates of van Rheede et al. [2]

	Soft maximum = 185 MYA – based on estimated age of Monotremata-Theria split assumed here
**Eulipotyphla-Xenarthra**	Hard minimum = 58.5 MYA – age of oldest known xenarthran, *Riostegotherium yanei* [10]
	Mean = 96 MYA – lower end (lower bound of 95% confidence interval) of molecular date of Murphy et al. [11]
	Soft maximum = 125.4 MYA – age (upper bound) of oldest eutherian, *Eomaia scansoria* [8]
**Crown marsupial radiation**	Hard minimum = 58.5 MYA – oldest known crown-marsupials from the late Palaeocene Itaborai fauna [12-15]
	Mean = 75.4 MYA – lower end (1 standard deviation less than the point estimate) of molecular date of Beck [16]
	Soft maximum = 98.5 MYA – age (upper bound) of oldest stem-marsupial, *Kokopellia juddi* [17]
**Crown australidelphian radiation**	Hard minimum = 23.03 MYA – age (lower bound) of oldest undoubted australidelphians [4,5]
	Mean = 63.5 MYA – lower end (1 standard deviation less than the point estimate) of molecular date of Beck [16]
	Soft maximum = 71.2 MYA – age (lower bound) of the Alamitian fauna of South America, in which marsupials appear to be completely absent; there is no evidence of australidelphians amongst the numerous Late Cretaceous metatherians known

	from North America, suggesting the Australidelphia did not radiate until marsupials reached South America, at the earliest
***Thylacinus*-dasyurids**	Hard minimum = 23.03 MYA – age (lower bound) of oldest known thylacinid, *Badjcinus turnbulli* [18]<br><br>Mean = 23.8 MYA - lower end (1 standard deviation less than the point estimate) of molecular date of Beck [16]<br><br>Soft maximum = 54.65 MYA - age (upper bound) of the Tingamarra Local Fauna [19], in which there is no evidence of either thylacinids or dasyurids
**Dasyurinae-Phascogalinae**	Hard minimum = 4.45 MYA – age (lower bound) of the oldest known crown dasyurine, cf. *Dasyurus* sp. [20], and the oldest known phascogaline, *Antechinus* sp. [20,21]<br><br>Mean = 10.0 MYA - lower end (1 standard deviation less than the point estimate) of molecular date of Beck [16]<br><br>Soft maximum = 23.03 MYA – age (upper bound) of oldest stem-dasyurid, *Barinya wangala* [22]
Dasyurus-Dasyuroides	Hard minimum = 4.45 MYA – age (lower bound) of cf. *Dasyurus* sp. [20]<br><br>Mean = 6.3 MYA - lower end (1 standard deviation less than the point estimate) of molecular date of Beck [16]<br><br>Soft maximum = 23.03 MYA – age (upper bound) of oldest stem-dasyurid, *Barinya wangala* [22]

**Didelphini-*Monodelphis***	Hard minimum = 11.8 MYA – age (lower bound) of oldest known undoubted crown didelphid, *Micoureus laventicus* [23]; *Micoureus* is more closely related to *Monodelphis* than to didelphins [24,25]<br><br>Mean = 25.3 - lower end (1 standard deviation less than the point estimate) of molecular date of Steiner et al. [25]<br><br>Soft maximum = 60.4 MYA – age (upper bound) of Middle Palaeocene Tiupampa fauna (following Gradstein et al. [26]), at which didelphids appear to be entirely absent
***Echymipera*-Peramelinae**	Hard minimum = 3.53 MYA – age of cf. *Peroryctes* spp. [20], the oldest known peroryctines<br><br>Mean = 10.2 MYA – lower end (1 standard deviation less than the point estimate) of molecular date of Beck [16]<br><br>Soft maximum = 23.03 MYA – age (upper bound) of *Yarala burchfieldi*, the oldest known stem-peramelemorphian [27,28]
**Phalangerida-Vombatiformes**	Hard minimum = 23.03 MYA – lower age estimate of oldest phalangeridans and vombatiforms [4,5]<br><br>Mean = 53.7 MYA - lower end (1 standard deviation less than the point estimate) of molecular date of Beck [16]<br><br>Soft maximum = 65 MYA, following Raterman et al. [29]

**Crown phalangeridan radiation**	Hard minimum = 23.03 MYA – oldest known petauroids, burramyids, phalangerids, and macropodoids [4,5]<br><br>Mean = 47.9 MYA - lower end (1 standard deviation less than the point estimate) of molecular date of Beck [16]<br><br>Soft maximum = 54.65 MYA – upper age estimate of Tingamarra fauna [19], in which there is no evidence of phalangeridans
Phascolarctos-Vombatus	Hard minimum = 23.03 MYA – oldest known phascolarctids and vombatoids [4,5]<br><br>Mean = 37.4 MYA - lower end (1 standard deviation less than the point estimate) of molecular date of Beck [16]<br><br>Soft maximum = 54.65 MYA – upper age estimate of Tingamarra fauna [19], in which there is no evidence of vombatiforms
**Crown-macropodid radiation**	Hard minimum = 4.45 MYA – lower age estimate of *Dorcopsis wintercookorum* and *Thylogale ignis*, the oldest known crown macropodids [30]<br><br>Mean = 12.6 MYA - lower end (1 standard deviation less than the point estimate) of molecular date of Beck [16]<br><br>Soft maximum = 23.03 MYA – upper age estimate of oldest known macropodids [4,5]
Phalanger-Trichosurus	Hard minimum = 23.03 MYA – lower age estimates of oldest trichosurins [31]

	Mean = 24.9 MYA – lower end (1 standard deviation less than the point estimate) of molecular date of Beck [16]
	Soft maximum = 54.65 MYA – upper age estimate of Tingamarra fauna [19], in which there is no evidence of phalangerids
**Petauridae-Pseudocheiridae**	Hard minimum = 23.03 MYA – lower age estimate of oldest known petaurids and pseudocherids [4,5]
	Mean = 29.3 MYA – lower end (1 standard deviation less than the point estimate) of molecular date of Beck [16]
	Soft maximum = 54.65 MYA – upper age estimate of Tingamarra fauna [19], in which there is no evidence of petauroids

REFERENCES

1. Benton MJ, Donoghue PCJ (2007) Paleontological evidence to date the tree of life. Molecular Biology and Evolution: 26-53.
2. van Rheede T, Bastiaans T, Boone DN, Hedges SB, de Jong WW, et al. (2006) The platypus is in its place: nuclear genes and indels confirm the sister group relation of monotremes and therians. Molecular Biology and Evolution 23: 587-597.
3. Kielan-Jaworowska Z, Cifelli RL, Luo Z-X (2004) Mammals from the age of dinosaurs: origins, evolution, and structure. New York: Columbia University Press.
4. Long JA, Archer M, Flannery TF, Hand SJ (2002) Prehistoric mammals of Australia and New Guinea : one hundred million years of evolution. Sydney: UNSW Press.
5. Archer M, Arena R, Bassarova M, Black K, Brammall J, et al. (1999) The evolutionary history and diversity of Australian mammals. Australian Mammalogy 21: 1-45.

6. Bininda-Emonds ORP, Cardillo M, Jones KE, MacPhee RDE, Beck RMD, et al. (2007) The delayed rise of present-day mammals. Nature 446: 507-512.

7. Rich TH, Vickers-Rich P, Trusler P, Flannery TF, Cifelli RL, et al. (2001) Monotreme nature of the Australian Early Cretaceous mammal *Teinolophos trusleri*. Acta Palaeontologica Polonica 46: 113-118.

8. Ji Q, Luo Z-X, Yuan C-X, Wible JR, Zhang J-P, et al. (2002) The earliest known eutherian mammal. Nature 416: 816-822.

9. Luo Z-X, Ji Q, Wible JR, Yuan C-X (2003) An Early Cretaceous tribosphenic mammal and metatherian evolution. Science 302: 1934-1940.

10. Rose KD, Emry RJ, Gaudin T, J., Storch G (2005) Xenarthra and Pholidota. In: Rose KD, Archibald JD, editors. The rise of placental mammals. Baltimore and London: The John Hopkins University Press.

11. Murphy WJ, Pringle TH, Crider TA, Springer MS, Miller W (2007) Using genomic data to unravel the root of the placental mammal phylogeny. Genome Research 17: 413-421.

12. Oliveira EV, Goin FJ, Candela AM (1996) Un nuevo marsupial "pseudodiprotodonte" del Paleoceno Medio de Itaborai (Brasil). Consideraciones sobre el origen, radiación y heterocronía en los Paucituberculata. Ameghiniana 33: 468.

13. Szalay FS (1994) Evolutionary history of the marsupials and an analysis of osteological characters. Cambridge: Cambridge University Press. 481 p.

14. Ladevèze S (2004) Metatherian petrosals from the Late Paleocene of Itaboraí (Brazil), and their phylogenetic implications. Journal of Vertebrate Paleontology 24: 202-213.

15. Ladevèze S (2007) Petrosal bones of metatherian mammals from the Late Palaeocene of Itaboraí (Brazil), and a cladistic analysis of petrosal features in metatherians. Zoological Journal of the Linnean Society 150: 85-115.

16. Beck RMD (2008) A dated phylogeny of marsupials using a molecular supermatrix and multiple fossil constraints. Journal of Mammalogy 89: 175-189.

17. Cifelli RL, de Muizon C (1997) Dentition and jaw of *Kokopellia juddi*, a primitive marsupial or near-marsupial from the Medial Cretaceous of Utah. Journal of Mammalian Evolution 4: 241-258.

18. Muirhead J, Wroe S (1998) A new genus and species, *Badjcinus turnbulli* (Thylacinidae: Marsupialia), from the late Oligocene of Riversleigh, northern Australia, and an investigation of thylacinid phylogeny. Journal of Vertebrate Paleontology 18: 612-626.
19. Godthelp H, Archer M, Cifelli RL, Hand SJ, Gilkeson CF (1992) Earliest known Australian Tertiary mammal fauna. Nature 356: 514-516.
20. Turnbull WD, Lundelius Jr. EL, Archer M (2003) Dasyurids, perameloids, phalangeroids, and vombatoids from the Early Pliocene Hamilton Fauna, Victoria, Australia. Bulletin of the American Museum of Natural History 279: 513-540.
21. Archer M (1982) Review of the dasyurid (Marsupialia) fossil record, integration of data bearing on phylogenetic interpretation, and suprageneric classification. In: Archer M, editor. Carnivorous marsupials. Mosman, New South Wales: Royal Zoological Society of New South Wales. pp. 397-443.
22. Wroe S (1999) The geologically oldest dasyurid (Marsupialia), from the Miocene Riversleigh, northwestern Queensland. Palaeontology 42: 1-27.
23. Goin FJ (1997) New clues for understanding Neogene marsupial radiations. In: Kay RF, Madden RH, Cifelli RL, Flynn JJ, editors. Vertebrate paleontology in the Neotropics: the Miocene fauna of La Venta, Colombia. Washington: Smithsonian Institution Press. pp. 187-206.
24. Jansa SA, Voss RS (2005) Phylogenetic relationships of the marsupial genus *Hyladelphys* based on nuclear gene sequences and morphology. Journal of Mammalogy 86: 853-865.
25. Steiner C, Tilak M-k, Douzery EJP, Catzeflis FM (2005) New DNA data from a transthyretin nuclear intron suggest an Oligocene to Miocene diversification of living South America opossums (Marsupialia: Didelphidae). Molecular Phylogenetics and Evolution 35: 363–379.
26. Gradstein F, Ogg J, Smith A, editors (2004) A geologic time scale 2004. Cambridge: Cambridge University Press.
27. Muirhead J (2000) Yaraloidea (Marsupialia, Peramelemorphia), a new superfamily of marsupial and a description and analysis of the cranium of the Miocene of *Yarala burchfieldi*. Journal of Paleontology 74: 512-523.
28. Muirhead J, Filan S (1995) *Yarala burchfieldi* (Peramelemorphia) from Oligo-Miocene deposits of Riversleigh, northwestern Queensland. Journal of Paleontology 69: 127-134.

29. Raterman D, Meredith RW, Ruedas LA, Springer MS (2006) Phylogenetic relationships of the cuscuses and brushtail possums (Marsupialia:Phalangeridae) using the nuclear gene BRCA1. Australian Journal of Zoology 54: 353-361.
30. Flannery TF, Rich TH, Turnbull WD, Lundelius Jr. EL (1992) The Macropodoidea (Marsupialia) of the early Pliocene Hamilton local fauna, Victoria, Australia. Fieldiana 25: 1-37.
31. Crosby KML (2004) Studies in the diversity and evolution of phalangeroid possums (Marsupialia; Phalangerida; Phalangeroidea) [Unpublished Ph.D. dissertation]. Sydney, Australia: University of New South Wales.
