# Supplementary material for: Australia's Oldest Marsupial Fossils and their Biogeographical Implications
Source: PLoS One. 2008 Mar 26;3(3):e1858. doi: 10.1371/journal.pone.0001858 (PMC2267999; doi:10.1371/journal.pone.0001858)
Supplement: Table S1 — Measurements of maximum petrosal length and M2 mesiodistal length for a range of extant and fossil marsupials (fossil taxa are indicated by †). Measurements for Djarthia murgonensis assume that the Tingamarran metatherian petrosals QM F36397, F36393 and F32322 (illustrated in Figure 1) are referrable to that taxon. No petrosal measurement is available for Thylacotinga bartholomaii because the petrosal of this taxon is currently unknown. (0.02 MB PDF) [file pone.0001858.s005.pdf]

Species	Family	specimen number	Maximum petrosal length (mm)	M2 mesiodistal length (mm)
Antechinus stuartii	Dasyuridae	UNSW 145 (left)	7.9	1.83
Antechinus stuartii	Dasyuridae	UNSW 145 (right)	7.8	1.98
Barinya wangala†	Dasyuridae	QM F31408 (left)	16.2	7.7
Barinya wangala†	Dasyuridae	QM F31408 (right)	16.2	7.5
Barinya wangala†	Dasyuridae	QM F31409	14.5	8.1
Dasyuroides byrnei	Dasyuridae	AR118 (left)	18	6.4
Dasyuroides byrnei	Dasyuridae	AR118 (right)	18.3	6.9
Dasyurus maculatus	Dasyuridae	J.8754 (right)	27.7	15.2
Dasyurus sp.	Dasyuridae	AR 6523 (left)	19.6	10.7
Dasyurus sp.	Dasyuridae	AR 6523 (right)	19.8	11.1
Dasyurus viverrinus	Dasyuridae	AR6521 (right)	20	12
Murexia sp.	Dasyuridae	no data (left)	11.7	6.7
Murexia sp.	Dasyuridae	no data (right)	10.8	6.9
Phascogale tapoatafa	Dasyuridae	AR 17644 (left)	17.6	7.4
Phascogale tapoatafa	Dasyuridae	AR 17644 (right)	15.7	7.8
Phascogale tapoatafa	Dasyuridae	no data (left)	16.3	7.1
Sarcophilus harrisii	Dasyuridae	UNSW 2414 (left)	39.8	25.9
Sarcophilus harrisii	Dasyuridae	UNSW 2414 (right)	41.9	27.5
Sminthopsis murina	Dasyuridae	AR1571 (left)	3.6	1.65
Sminthopsis murina	Dasyuridae	AR1571 (right)	3.4	1.67

Didelphis marsupialis	Didelphidae	Zool 248 (left)	25.5	11.6
Didelphis marsupialis	Didelphidae	Zool 248 (right)	26.2	12.6
Didelphis marsupialis	Didelphidae	Zool 401 (left)	22.7	13.7
Didelphis marsupialis	Didelphidae	Zool 401 (right)	20.7	14.5
Didelphis sp.	Didelphidae	no data (left)	22.6	13.8
Didelphis sp.	Didelphidae	no data (right)	24.6	13.9
Marmosa pusilla	Didelphidae	no data (left)	4.15	1.8
Marmosa pusilla	Didelphidae	no data (right)	4	1.91
Marmosa sp.	Didelphidae	no data (left)	3.72	1.6
Marmosa sp.	Didelphidae	no data (right)	3.74	1.55
Aeprymnus rufus	Macropodidae	no data (left)	22	14
Dromiciops australis	Microbiotheriidae	AR00452 (left)	4.5	1.55
Dromiciops australis	Microbiotheriidae	AR00452 (right)	4.8	1.6
Dromiciops sp.	Microbiotheriidae	no data (left)	9.6	1.65
Dromiciops sp.	Microbiotheriidae	no data (right)	9.8	1.57
Perameles nasuta	Peramelidae	UNSWZ 32 (left)	17.8	8.6
Perameles nasuta	Peramelidae	UNSWZ 32 (right)	16.5	8.9
Echymipera kalubu	Peroryctidae	AR 7644 (left)	10.9	7.9
Echymipera kalubu	Peroryctidae	AR 7644 (right)	11	8.4
Echymipera kalubu	Peroryctidae	AR 7248 (left)	15.3	7.7

Echymipera kalubu	Peroryctidae	AR 7248 (right)	12.4	8
Peroryctes raffrayana	Peroryctidae	R157 (left)	14	7.6
Peroryctes raffrayana	Peroryctidae	R157 (right)	12.8	8.2
Petaurus australis	Petauridae	UNSWZ 455 (left)	17.7	6
Petaurus australis	Petauridae	UNSWZ 455 (right)	17.1	6
Spilocuscus maculatus	Phalangeridae	AR11383 (right)	25.1	15.9
Trichosurus sp.	Phalangeridae	no data (right)	28.1	11
Nimbacinus dicksoni†	Thylacinidae	QM F36357 (left)	25.1	18.7
Yalara burchfieldi†	Yaralidae	QM F16860 (right)	3.45	1.64
Djarthia murgonensis†	unknown	QM F36397, F36393 and F32322 (petrosal measurement); QM F31458 (M2 measurement)	3.8	1.81
Thylacotinga bartholomaii†	unknown	QM F16835	-	5.58
