# Supplementary material for: Australia's Oldest Marsupial Fossils and their Biogeographical Implications
Source: PLoS One. 2008 Mar 26;3(3):e1858. doi: 10.1371/journal.pone.0001858 (PMC2267999; doi:10.1371/journal.pone.0001858)
Supplement: Table S2 — Measurements of lower ankle joint width (taken from the calcaneus) and M2 mesiodistal length for a range of extant marsupials, plus Djarthia murgonensis and Thylacotinga bartholomaii. Measurements for D. murgonensis assume that the Tingamarran metatherian calcanea QM F52747 (illustrated in Figure 2), F52748 and F 52749 are referable to that taxon. No lower ankle joint width measurement is available for T. bartholomaii, as the calcaneus of this taxon is currently unknown. (0.01 MB PDF) [file pone.0001858.s006.pdf]

Species	Family	specimen number	lower ankle joint width (mm)	M2 mesiodistal length (mm)
Antechinus stuarti	Dasyuridae	AR1674 (right)	1.46	2.04
Dasyuroides byrnei	Dasyuridae	AR9360 (left)	6.1	6.4
Sminthopsis murina	Dasyuridae	AR1571 (right)	1.42	1.73
Didelphis sp.	Didelphidae	no data (right)	16	14.7
Marmosa sp.	Didelphidae	no data (right)	1.21	1.79
Thylamys pusilla	Didelphidae	no data (right)	1.22	1.87
Dromiciops sp.	Microbiotheriidae	no data (right)	1.5	1.7
Isoodon sp.	Peramelidae	no data (left)	13	9.5
Isoodon macrourus	Peramelidae	no data (right)	9.3	8.9
Peroryctes sp.	Peroryctidae	No. 33 (right)	3.4	5.25
Petaurus sp.	Petauridae	no data (right)	6.8	5
Djarthia murgonensis†	unknown	QM F52747 (lower ankle joint width); QM F31458 (M2 mesiodistal length)	1.4	1.81
Djarthia murgonensis†	unknown	QM F52748 (lower ankle joint width); QM F31458 (M2 mesiodistal length)	1.65	1.81
Djarthia murgonensis†	unknown	QM F52749 (lower ankle joint width); QM F31458 (M2 mesiodistal length)	1.75	1.81

Thylacotinga bartholomaii†	unknown	QM F16835	-	5.58
