# Supplementary material for: Australia's Oldest Marsupial Fossils and their Biogeographical Implications
Source: PLoS One. 2008 Mar 26;3(3):e1858. doi: 10.1371/journal.pone.0001858 (PMC2267999; doi:10.1371/journal.pone.0001858)
Supplement: Table S3 — Molecular divergence dates within marsupials as calculated by BEAST assuming an uncorrelated lognormal relaxed clock. Dates were calculated using the supermatrix of Beck [6], including (‘full’) and excluding (‘no mt3’) the third codon positions of mitochondrial protein-coding genes. Node numbers correspond to the phylogeny in Figure S3. Point estimates and 95% confidence intervals are given for each node. (0.01 MB PDF) [file pone.0001858.s007.pdf]

	'full'		'no mt3'	
Node	date (Ma)	95% CI (Ma)	date (Ma)	95% CI (Ma)
1	183.72	169.22-200.9	186.59	171.76-206.51
2	97.91	80.5-115.63	100.25	79.43-123.65
3	145.49	117.99-170.17	157.58	134.38-182.55
4	79.56	71.74-89.12	79.69	70.48-90.46
5	36.89	28.25-45.02	37.69	27.3-49.13
6	74.13	66.81-81.74	75.09	66.19-84.32
7	65.5	59.24-71.77	65.04	59.21-71.28
8	63.84	57.64-70.11	62.6	56.76-68.68
9	53.65	49.16-58.37	53.42	48.56-58.84
10	47.3	42.65-51.4	46.02	40.81-50.7
11	43.48	38.47-47.64	42.5	37.81-48.28
12	14.23	10.79-18.58	13.11	9.55-16.96
13	11.41	8.1-15.61	10.54	7.27-13.18
14	36.94	30.33-44.29	35.49	29.85-41.63
15	25.66	23.21-29.39	25.71	23.25-28.88
16	28.29	24.02-33.07	29.77	24.67-35.45
17	35.73	29.74-44.91	36.13	28.76-42.42
18	61.39	54.66-67.11	60.01	54.15-65.53
19	57.46	51.67-63.7	56.85	51.09-62.93
20	33.12	27.75-39.04	28.86	23.76-33.84
21	14.85	11.3-17.98	14.36	11.21-17.94
22	10.7	7.49-13.97	9.99	7.16-13.12
23	13.64	9.29-18.71	12.8	9.1-16.47
24	46.14	29.95-62.41	43.05	29.45-68.29
