# Supplementary figures and images for: Australia's Oldest Marsupial Fossils and their Biogeographical Implications
Source: PLoS One. 2008 Mar 26;3(3):e1858. doi: 10.1371/journal.pone.0001858 (PMC2267999; doi:10.1371/journal.pone.0001858)

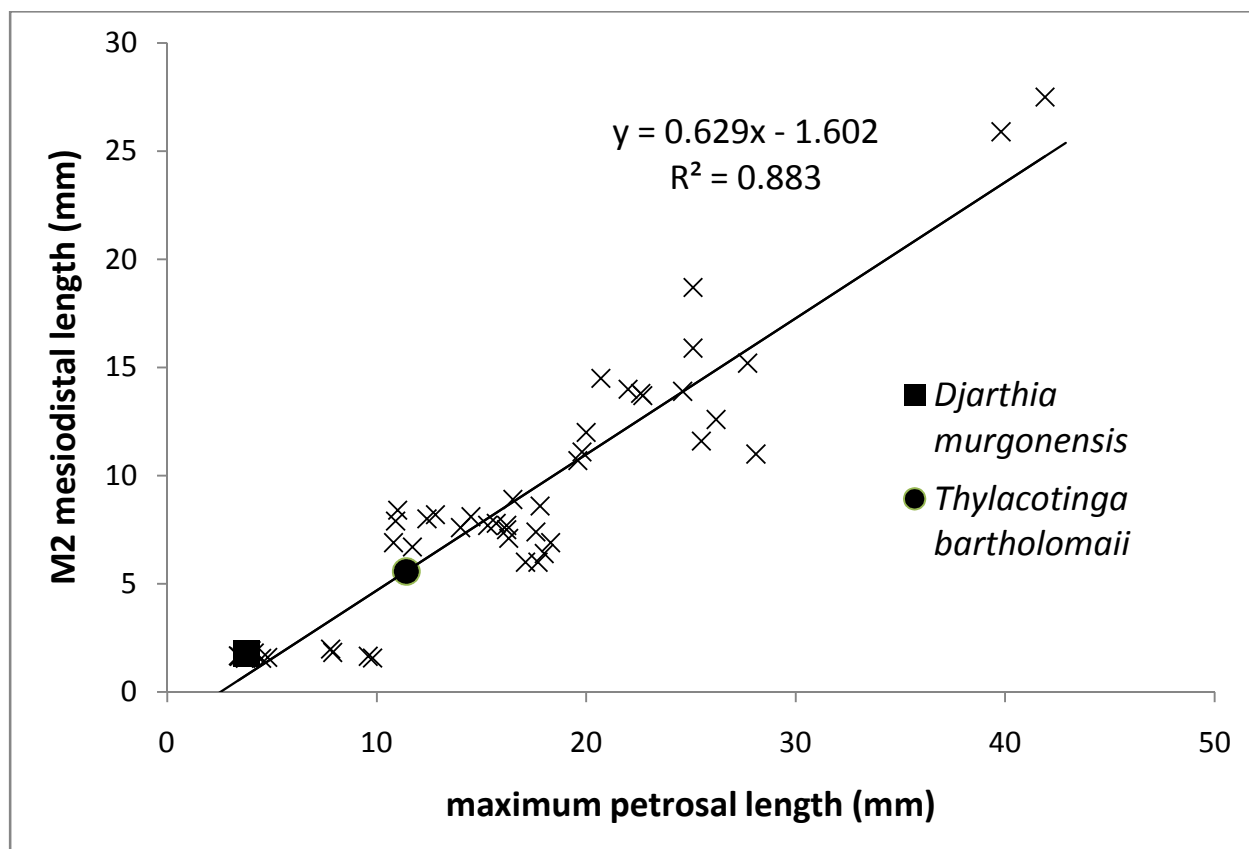

Supplement: Figure S1 — Plot of M2 mesiodistal length against maximum petrosal length for the specimens listed in Table S1. Specimens of Djarthia murgonensis and Thylacotinga bartholomaii are identified by squares and circles respectively. The predicted maximum petrosal length for T. bartholomaii was calculated according to the equation for the line of best fit. (0.04 MB PDF) [file pone.0001858.s008.pdf]

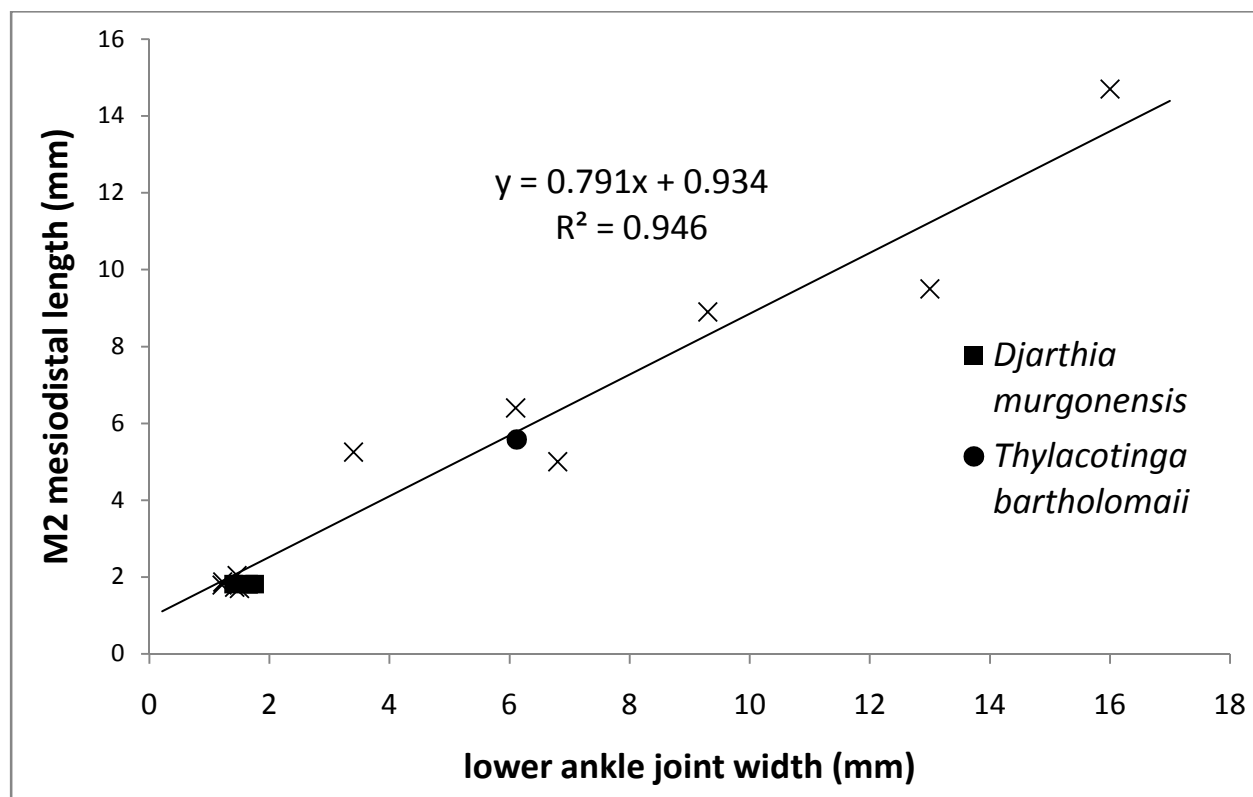

Supplement: Figure S2 — Plot of M2 mesiodistal length against lower ankle joint width for the specimens listed in Table S2. Specimens of Djarthia murgonensis and Thylacotinga bartholomaii are identified by squares and circles respectively. Predicted lower ankle joint width for T. bartholomaii was calculated according to the equation for the line of best fit. (0.04 MB PDF) [file pone.0001858.s009.pdf]
